# Advanced Footwear Technology in Distance Running: What We Know, What We Don't Know, and Future Directions

**DOI:** 10.1002/ejsc.70266

**Published:** 2026-10-12

**Authors:** Bas Van Hooren, Benedicte Vanwanseele, Emily Matijevich, Thomas Gronwald, Marlene Giandolini, Brian Hanley, Andrew M. Jones, Guillaume Rao, Christopher Napier, Hannah Rice, Anton Arndt, Karsten Hollander, Cedric Morio, Laurent Malisoux

**Affiliations:** ^1^ Department of Nutrition and Movement Sciences NUTRIM Institute of Nutrition and Translational Research in Metabolism Maastricht University Medical Centre+ Maastricht the Netherlands; ^2^ Department of Movement Sciences Human Movement Biomechanics Research Group Katholieke Universiteit Leuven Leuven Belgium; ^3^ Leuven Institute of Sports Science Leuven Belgium; ^4^ Faculty of Kinesiology Human Performance Laboratory University of Calgary Calgary Alberta Canada; ^5^ Institute of Interdisciplinary Exercise Science and Sports Medicine MSH Medical School Hamburg Hamburg Germany; ^6^ Research and Sciences Lab Salomon SAS Annecy France; ^7^ Carnegie School of Sport Leeds Beckett University Leeds UK; ^8^ Department of Public Health and Sport Sciences University of Exeter Medical School Exeter UK; ^9^ Aix Marseille Univ CNRS ISM Marseille France; ^10^ Department of Biomedical Physiology & Kinesiology Simon Fraser University Vancouver British Columbia Canada; ^11^ Department of Physical Performance Norwegian School of Sport Sciences Oslo Norway; ^12^ Department of Physiology, Nutrition and Biomechanics The Swedish School of Sport and Health Sciences Stockholm Sweden; ^13^ Department CLINTEC Karolinska Institute Stockholm Sweden; ^14^ Decathlon SportsLab Decathlon SE Lille France; ^15^ Department of Precision Health Luxembourg Institute of Health Strassen Luxembourg

**Keywords:** individual response, injury risk, performance, running, running economy, super shoes

## Abstract

Over the past decade, running shoes have undergone substantial innovation with the emergence of Advanced Footwear Technology (AFT). Shoes with AFT are typically characterized by highly compliant (deformable) and resilient (high energy return) midsole foams, embedded curved stiffening elements, low mass, and a rockered longitudinal geometry. This expert statement aims to synthesize established knowledge, remaining uncertainties and key directions for future research on AFT in middle‐ and long‐distance running. Current literature demonstrates robust improvements in running economy and performance with AFT, with reported metabolic savings of approximately 2%–4%, and corresponding performance improvements of 1%–3%. These benefits likely arise from a combination of “acute” and possibly “long‐term” mechanisms. Proposed acute mechanisms include decreased muscle force production due to higher positive work from energy‐returning foams, lower muscle force production to accommodate mechanical running demands due to compliant midsoles, and improved mechanical efficiency associated with greater longitudinal bending stiffness and curved geometry. Importantly, AFT features have a synergistic effect, with the relative importance of each mechanism differing depending on the context. Emerging evidence also suggests potential reductions in muscle damage/inflammation and soreness, which could support better physiological resilience, and higher training volumes/quality. From a biomechanical injury risk perspective, AFT can redistribute loading, potentially increasing metatarsal and tarsal stress while reducing tibia and possibly Achilles tendon loading. However, injury risk associated with AFT use remains equivocal, with limited high‐quality data. Notably, substantial inter‐individual variability has been observed, influencing both running economy and potential injury risk although the determinants of this variability remain unclear.

## Highlights

- Advanced footwear technology (AFT) improves running economy by approximately 2%–4% and running performance by 1%–3%.
- AFT benefits arise from interacting acute mechanisms involving midsole compliance and resilience, longitudinal stiffness, rocker geometry, and reduced shoe mass, but the importance of each mechanism differs depending on the context.
- AFT can redistribute loading, potentially increasing metatarsal and tarsal stress while reducing tibia and possibly Achilles tendon loading; however, its effects on injury risk remain uncertain and may vary substantially between individuals.
- Future research should identify determinants of individual responses and clarify the long‐term effects of AFT on performance and injury risk.

## 1 Introduction

Running shoe technology has evolved from relatively simple foam midsoles attached to an upper construction into highly engineered systems designed both to improve performance and mitigate injury risk. Early innovations focussed primarily on adding cushioning, reducing mass, and providing stability, but their effects on performance and injury prevention were often inconsistent and modest (Malisoux and Theisen 2020; Agresta et al. 2022). By contrast, over the last 10 years, running shoes have seen considerable advancements with the introduction of Advanced Footwear Technology (AFT) (Burns and Joubert 2024; Frederick 2022). AFT shoes are characterized by highly compliant (deformable) and resilient (energy‐returning) midsole foams, embedded curved stiffening elements, a low mass, and a rocker (curved) longitudinal geometry (Figure 1). Since the widespread adoption of shoes featuring AFT in late 2016, substantial improvements in middle‐ and long‐distance running performance have been reported, in both road and track racing (Rodrigo‐Carranza et al. 2021, 2022; Langley et al. 2023), with less conclusive effects for trail running (Corbí‐Santamaría et al. 2025). The effects of AFT on running injuries are less clear. Further, the mechanisms by which AFT improves performance and influences injury risk remain debated. Similarly, the effects of AFT on performance and injury across varied contexts, as well as the factors underlying inter‐individual variability in response to AFT, remain unclear.

**FIGURE 1:**
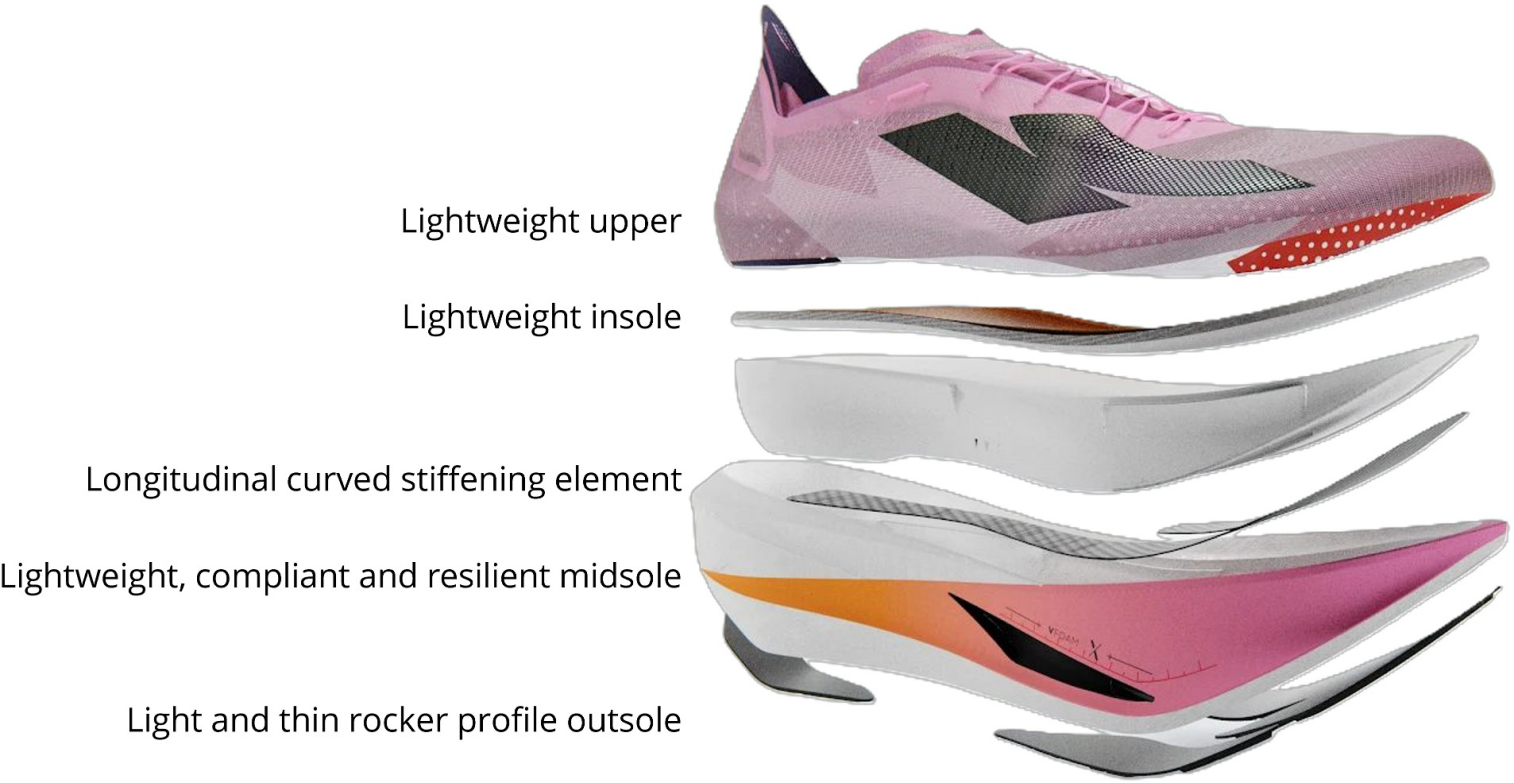
Features of advanced footwear technology.

Given the many unknown factors about AFT in relation to performance and injury risk, the purpose of this expert statement is to synthesize the current evidence on AFT in middle‐ and long‐distance running. Specifically, we address what we know and do not yet understand and propose future directions for research.

## 2 Effects of AFT on Running Performance

Various sources of evidence strongly indicate that AFT improves middle‐ and long‐distance running performance by an average of ∼1%–3%, depending on the race distance (Rodrigo‐Carranza et al. 2021, 2022; Langley et al. 2023; Bermon et al. 2021). Analyses of elite road and track races before and after widespread AFT adoption show significant reductions in finishing times, increased frequency of record‐setting performances, and a broader range of high‐level performances (as shown by improved top 100 season's best times) in both males and females and across a wide range of competition distances. These robust observations align with controlled studies demonstrating improvements of ∼1.1%–2.5% in 3–3.5 km time‐trial performance (Hebert‐Losier et al. 2022; Nielsen, Heyde, et al. 2022). However, although males and females typically show similar improvements in running economy (RE) with AFT (Seglina et al. 2026; Barnes and Kilding 2019; Joubert et al. 2023; Riedl et al. 2024; Matties and Rowley 2025; Xiao et al. 2026; Stephen et al. 2025; Perry et al. 2025), females experience a greater performance benefit than males (∼1.7%–4% vs. ∼0.6%–1.7%, respectively) (Bermon et al. 2021; Willwacher et al. 2024; Needles and Grabowski 2024; Maruo and Takezawa 2026). This may at least partly reflect the slower running speed of females at which positive effects on RE more directly translate into performance benefits (Kipp et al. 2019). Indeed, energy expenditure increases curvilinearly with running speed, and faster running also requires additional energy to overcome greater air resistance. Therefore, given metabolic savings translate into proportionally smaller performance gains, typically only about half to two‐thirds as large in terms of time savings (Kipp et al. 2019; Hoogkamer et al. 2016), such that females will typically experience greater performance benefits for a given RE improvement (Bermon et al. 2021; Willwacher et al. 2024; Needles and Grabowski 2024; Maruo and Takezawa 2026). Nevertheless, further research should explicitly explore if speed explains this effect, and if other factors such as running biomechanics, and anthropometrics also explain the greater relative performance benefit observed in females.

Beyond road running, AFT features have also been incorporated into middle‐ and long‐distance track spikes since 2019 and have been reported to improve RE by ∼2% relative to non‐AFT spikes (Joubert et al. 2024; Needles et al. 2026). However, ascertaining how RE (and other) benefits of AFT spikes translate to improved track race performance is less straightforward than for road shoes because of the larger nonoxidative energy contribution in middle‐distance events. Current research indicates a robust performance‐enhancing effect (1%–1.5%) of AFT spikes on track running performance (Willwacher et al. 2024; Needles and Grabowski 2024; Rodrigo‐Carranza et al. 2025; Bertschy et al. 2025) in line with predicted performance benefits from metabolic savings (Joubert et al. 2024). Similar to road running, the benefit depends on running speed with smaller performance benefits at faster running speeds (Needles and Grabowski 2024). The smaller performance benefits in AFT spikes relative to the ∼2% performance benefit with road running shoes (Needles and Grabowski 2024; Hebert‐Losier and Pamment 2023) might reflect the typically higher speed in shorter track events, but also the reduced thickness of foam that can be incorporated into spikes as World Athletics (2026) regulations cap the maximum permitted thickness at 40 mm for road running events and 20 mm for track events.

## 3 How Does AFT Enhance Running Performance?

The current evidence suggests that AFT enhances running performance through a combination of acute and possibly long‐term effects (Figure 2). “Acute” effects include an improved RE, and possibly an improved physiological resilience due to less muscular damage and fatigue. “Long‐term” effects could include the potential for larger training volumes and higher training quality due to less damage and/or fatigue, leading to enhanced training adaptations and therefore, improved performance through an indirect “AFT effect”.

**FIGURE 2:**
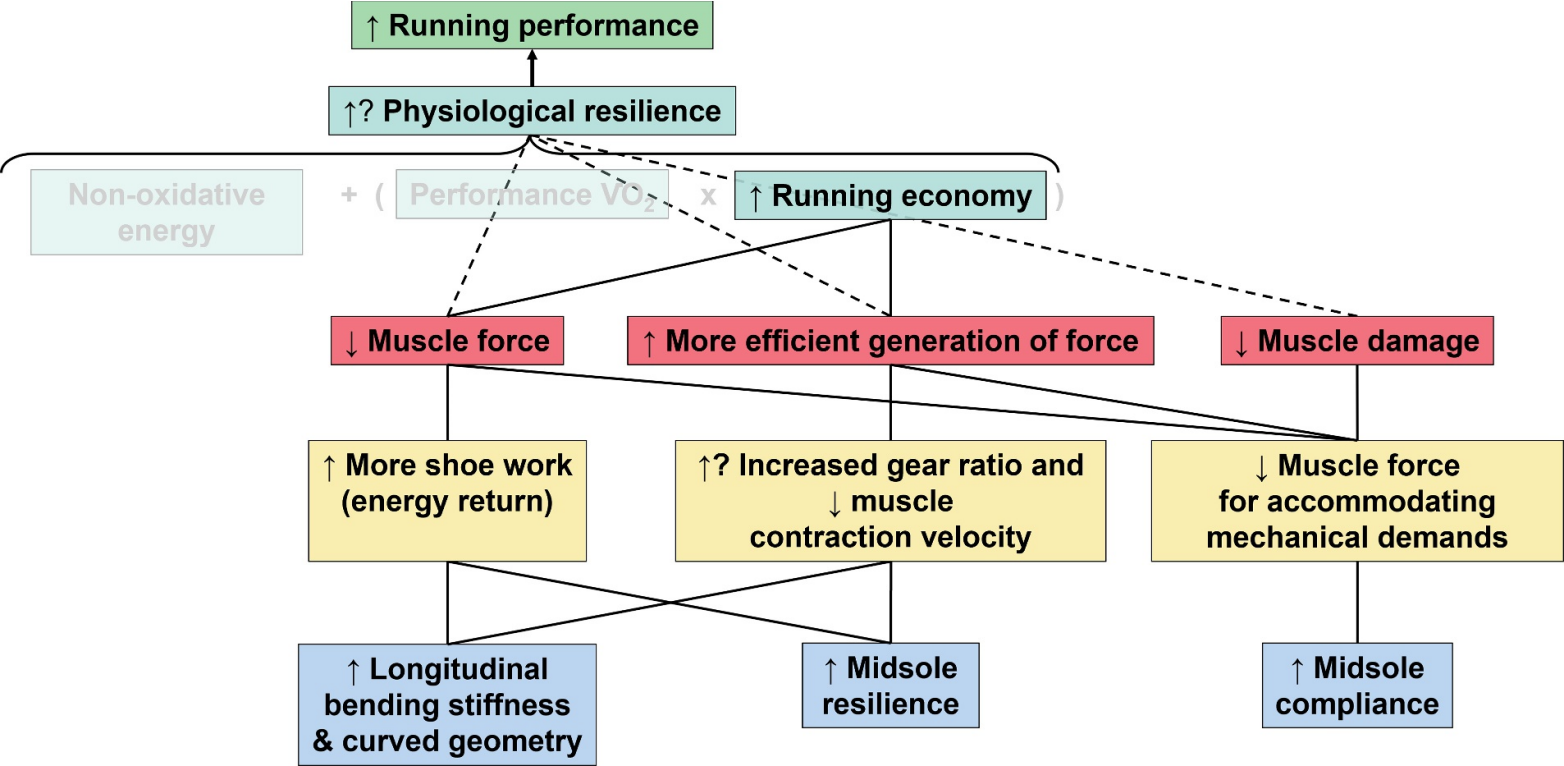
Conceptual model depicting how AFT features may alter running biomechanics, in turn affecting important physiological parameters known to influence running performance. Briefly, running performance is determined by the combination of (a) performance $\dot{V}O_{2}$ (which is related to the fraction of $\dot{V}O_{2}$ max that can be sustained for a given distance), (b) nonoxidative energy contribution, and (c) RE (Joyner 1991). Furthermore, the extent to which these physiological parameters deteriorate during prolonged running (i.e., physiological resilience (Jones 2024)) is an additional important factor. AFT shoes do not directly enhance the nonoxidative energy contribution or performance $\dot{V}O_{2}$ (i.e., fraction of $\dot{V}O_{2}$ that can be sustained for the given distance), hence, the faded boxes. Instead, they may enhance RE by a) reducing muscle force production (i.e., negative and positive muscle work), b) allowing muscles to generate a given force with greater efficiency (i.e., reduction in the cost of generating force), and c) attenuating muscle damage during prolonged running (which in turn could improve the resilience of RE). The reduced oxygen or energy consumption around the lactate threshold can additionally spare nonoxidative capacity. The reduced muscle force production at a given running speed (in particular at the ankle plantar flexors) likely results from a higher shoe energy return. The higher shoe energy return in turn is primarily due to higher midsole resilience, although it may be further enhanced by interacting with the stiffening element. The midsole compliance, longitudinal bending stiffness, and rocker shoe geometry may also redistribute the muscle force requirements to more efficient muscles (e.g., less knee extensor force and ankle power output) and might allow muscles to produce a given amount of force more efficiently, for example, by reducing their concentric contraction velocity. Finally, midsole compliance may reduce the muscular force normally required for accommodating the mechanical demands of body weight support, which both directly reduces energy cost and also attenuates the deterioration of RE during prolonged running by reducing muscle damage.

Regarding the acute beneficial effect on RE, meta‐analytic evidence indicates that AFT reduces the oxygen and energy cost of running—here collectively referred to as RE—by approximately 2%–4% on average (Xiao et al. 2026; Knopp et al. 2023). However, the magnitude varies across shoe models (Joubert et al. 2024; Joubert and Jones 2022; Denis et al. 2024; Nielsen, Franch, et al. 2022; Kuzmeski et al. 2025), individuals (Seglina et al. 2026; Knopp et al. 2023; Van Hooren et al. 2025; Hoogkamer et al. 2018; Heyde et al. 2022; Bidois et al. 2026), and testing protocols/conditions (Knopp et al. 2023; Barrons et al. 2024; Askew et al. 2026). For example, although most athletes benefit from AFT shoes (Van Hooren et al. 2025; Hoogkamer et al. 2018; Heyde et al. 2022; Bidois et al. 2026; Barrons et al. 2024; Beltrami et al. 2025), some athletes may experience no benefit, or even experience worse RE in certain AFT shoes compared to a control shoe (Knopp et al. 2023), although negative responses are largely—but not completely—absent in studies averaging multiple measures (Figure 3); (Bidois et al. 2026; Werkhausen et al. 2024). With regard to testing protocols, the percent benefit of AFT on RE generally increases with faster running speeds (Xiao et al. 2026; Stephen et al. 2025; Knopp et al. 2023). This effect is, however, smaller than the increase in energy required to overcome greater air resistance at higher running speeds, such that the net performance benefit decreases at faster speeds. Because of this interaction, significant and relevant improvements in RE have been found both at slower speeds of 7.5–12 km h^−1^, typical for recreational runners (Hebert‐Losier et al. 2022; Joubert et al. 2023; Van Hooren et al. 2025; Werkhausen et al. 2024; Paradisis et al. 2023; Bolliger et al. 2026; Martinez et al. 2024), as well as at faster speeds of 16–20 km h^−1^ typical for better‐trained to (sub) elite runners (Barnes and Kilding 2019; Knopp et al. 2023; Joubert and Jones 2022; Hoogkamer et al. 2018; Hunter et al. 2019). Mechanistically, the improvements in RE observed with AFT could reflect reductions in muscular force production requirements, an increased efficiency by which the force is produced—for example by reducing fascicle shortening velocity or redistributing force production to more economical muscles— and, during prolonged running, also reduced muscle damage.

### 3.1 Greater Midsole Energy Return May Reduce Muscle Force Production to Accelerate the Body Center of Mass

From a biomechanical perspective, the metabolic cost of running is determined by the muscular force and resulting amount of mechanical work that must be produced to support, accelerate and redirect the body's centre of mass (COM) relative to the environment (external work) and the amount of work required to accelerate body segments relative to the COM (internal work) (Riddick and Kuo 2022). Although AFT does not reduce the external mechanical work of running (i.e., the runner must still generate the same vertical and horizontal impulses to move the COM forward), AFT can alter the distribution of this work (i.e., muscle force production), and thereby RE, in two ways.

First, AFT can reduce the internal mechanical work by slightly increasing the step length at a given speed and thereby lowering the mechanical work required to swing the legs. However, the increased step length has not been found to be associated with the benefit in RE (Perry et al. 2025; Joubert and Jones 2022; Van Hooren et al. 2025; Hoogkamer et al. 2018; Fohrmann et al. 2026), possibly because the lower internal work associated with larger step lengths might be counteracted by the higher external work due to greater horizontal braking and propulsive forces and larger vertical displacement (Lieberman et al. 2015). Thus, the potential gains in reduced internal work with AFT could be negated by greater external work, resulting in no clear RE advantage.

Second, AFT alters how the mechanical work is distributed between the body (muscles and tendons) and the shoes (Hoogkamer et al. 2019; Matijevich et al. 2025; Matijevich et al. 2023). Specifically, highly compliant and resilient midsole foams can store and return (by rebound) elastic energy during stance (Hoogkamer et al. 2018; Burns and Tam 2020; Flores et al. 2019; Worobets et al. 2014). Benchtop tests where a shoe is compressed with vertical force magnitudes similar to those during running show that AFT shoes typically return 80%–90% of stored compression mechanical work, compared with 55%–70% in non‐AFT shoes (Needles et al. 2026; Kuzmeski et al. 2025; Van Hooren et al. 2025; Hoogkamer et al. 2018; Bidois et al. 2026; Askew et al. 2026; Fohrmann et al. 2026; Matijevich et al. 2025; Kiesewetter et al. 2022). This higher energy return (positive shoe work) in AFT shoes may effectively allow part of the mechanical work required to displace the body COM to be performed by the shoe rather than by active muscle contraction. Indeed, running with AFT has been demonstrated to reduce the positive (and negative) work, power, or torque at the ankle and metatarsophalangeal (MTP) joints (Werkhausen et al. 2024; Martinez et al. 2024; Hoogkamer et al. 2019; Bruneau et al. 2026), amongst others, due to the greater positive work delivered by shoe energy return (Hofmann 2026). A putative decrease in the positive muscular mechanical work at one or more joints due to some work being performed by shoes may in turn reduce the metabolic energy expenditure.

The relatively high energy return capacity of AFT is primarily attributed to the rebound of midsole foam rather than the longitudinal stiffening element because the elastic energy stored and returned by the latter is estimated to be only 2% of the energy stored and returned by the midsole material (Hoogkamer et al. 2019). While the elastic energy stored and returned by the longitudinal stiffening element is therefore small, it may interact with the foam by distributing the compression across a larger area (i.e., a point‐elastic area is converted into an area‐elastic structure (Burns and Joubert 2024; Kram 2022)). This may, in turn, optimize energy storage and return of the midsole material (Burns and Joubert 2024). Cutting the plate would not completely remove this effect and this may therefore partly explain previous findings where cuts in the plate significantly reduced longitudinal bending stiffness but led to only a small nonsignificant worsening of RE (Healey and Hoogkamer 2022). However, the extent to which mechanical energy return from the midsole material translates to improved RE or performance remains unclear. Matijevich and colleagues (Matijevich et al. 2025) demonstrated that the greater energy storage and return capabilities of AFT observed during benchtop material testing did not translate directly to equivalent mechanical benefits during running, which may have been because the timing of energy storage and return was suboptimal. Similar findings were reported by Rodrigo‐Carranza, et al. (Rodrigo‐Carranza et al. 2026), who despite reporting a 13% point (300% increase) higher energy return (from 7.6 to 8.5 J) in their Asker C 35 (hardness measure, with lower values reflecting softer material) shoe compared with the Asker C 40 shoe, found that RE differed only by 0.27%. Although mechanical energy return *is* generally accepted to contribute to the metabolic savings in AFT (Hoogkamer et al. 2019; Hofmann 2026; Ferris et al. 2026; Ghanbari, Vienneau, et al. 2025), a given magnitude increase in energy return does therefore not result in equivalent reductions in metabolic cost.

Greater longitudinal bending stiffness can also reduce MTP‐negative work and ankle‐positive work (Ghanbari, Vienneau, et al. 2025), thereby benefiting energy cost. Specifically, during the stance phase of running, the MTP joint dorsiflexes from touchdown until toe‐off. Because it remains dorsiflexed through push‐off, it returns little to no energy and instead likely absorbs mechanical energy (i.e., dissipates it into heat). The greater longitudinal bending stiffness in AFT—and possibly the rockered shoe profile— limits dorsiflexion magnitude and velocity (Ghanbari, Vienneau, et al. 2025; Ortega et al. 2021), thereby decreasing the amount of mechanical energy lost at the MTP joint and reducing energy cost. Nevertheless, because of the greater relative metabolic contribution of the ankle plantar flexors compared with that of the MTP joint, and the approximately five times greater energy cost of positive versus negative work (Margaria 1976), the reduction in energy generation at the ankle joint due to greater midsole energy return may contribute more to overall metabolic savings than the reduction in MTP negative work due to a stiffening element. In support of this finding, Ghanbari, Vienneau, et al. (2025) found a greater role for midsole energy return than the presence of a plate in the improvement in RE with AFT. Similarly, reducing longitudinal bending stiffness by cutting the plate worsened (albeit nonsignificantly) running economy by a relatively small magnitude of 0.55%, suggesting an important role for other plate properties (Healey and Hoogkamer 2022).

Finally, the curved geometry of the shoe outsole may also influence RE. While two studies investigating the effects of an extrinsic rocker on running shoes reported no significant benefit on RE (Sobhani et al. 2014; Tankink et al. 2024), one of the studies reported a small (∼0.8%) nonsignificant reduction in energy cost along with a more proximal distribution of mechanical work when only modifying the rocker profile (Tankink et al. 2024). However, more research is required to assess the effects of the curved geometry in a complete AFT shoe. In this regard, some findings indicate that the curved geometry in AFT may reduce muscle work by the teeter‐totter effect, whereby the downward force in the anterior portion of the shoe during late stance creates an upward force in the posterior portion of the shoe (Subramanium et al. 2024), thereby lowering muscle work. However, further research is required to more fully explain this mechanism.

### 3.2 More Efficient Production of Muscle Force Production

By slightly changing the running technique, AFT can also redistribute the force production requirements to more efficient (i.e., with lower metabolic cost to produce a given magnitude of force) active or passive structures, or allow one or more muscles to operate at a more favorable position of the force–length–velocity relationship. For example, shoes with greater longitudinal bending stiffness can redistribute some of the mechanical positive work to the ankle and MTP joint instead of the knee joint (Ghanbari, Vienneau, et al. 2025; Cigoja et al. 2021; Cigoja et al. 2022), although this is not universally observed (Ortega et al. 2021). Moreover, running in shoes with increased longitudinal bending stiffness can delay the onset of the joint work redistribution from the ankle to the knee joint that can occur with fatigue (Cigoja et al. 2022; Nahan et al. 2025). Because the ankle extensors are often more efficient (i.e., requiring lower metabolic cost) for producing work at the slow plantar flexor fascicle velocities seen in running (Biewener and Daley 2007), these findings suggest that increasing longitudinal bending stiffness might both acutely enhance RE and improve physiological resilience during prolonged running.

An altered distribution of force production from the knee to the ankle muscles is also seen with highly compliant midsole foams. Specifically, highly compliant foams can prompt the motor system to increase leg stiffness, thereby reducing knee work, power and extension velocity without affecting ankle power (Petrella et al. 2026). The decreased knee extension velocity might, in turn, decrease the concentric contraction velocity of the knee extensors, thereby decreasing their energy cost (Swinnen et al. 2023; Fletcher and MacIntosh 2017). The effect of compliant midsoles on knee mechanics could explain why some studies observe a higher leg stiffness in AFT versus control shoes (Needles et al. 2026; Castellanos‐Salamanca et al. 2023). However, this is not universally observed (Martinez et al. 2024; Hoogkamer et al. 2019). Whether differences in knee mechanics are observed appears to depend, amongst other factors, on compliance differences between the AFT and reference shoe. For example, Hoogkamer et al. (2019) found no difference in the knee angle or moments in AFT versus non‐AFT shoes. However, the difference in displacement between the shoes under a 2000 N load was only 6 mm, whereas studies reporting differences in knee mechanics between surfaces observed displacements of up to 26 mm for a load of 2000 N (Kerdok et al. 2002). This smaller compliance difference between shoes versus surfaces could also explain other findings showing no differences in knee biomechanics in AFT versus control shoes (Martinez et al. 2024). Additionally, most studies do not account for shoe compliance when determining leg stiffness with the greater shoe compliance masking higher leg stiffness (Needles et al. 2026).

Further, some studies show that the higher longitudinal bending stiffness in AFT increases ground contact time (Ortega et al. 2021; Rodrigo‐Carranza, Hoogkamer, et al. 2024; Perrin et al. 2025) and decreases the ankle plantar flexion velocity (Stephen et al. 2025; Miyazaki et al. 2024) without changing ankle positive work or power (Stephen et al. 2025). This could, in turn, benefit RE by increasing the efficiency by which force is produced because the resulting slower plantar flexor fascicle velocity with stiffer curved longitudinal elements (Cigoja et al. 2021) (or generally in AFT (Hata et al. 2024)) may reduce energy cost (Swinnen et al. 2023; Fletcher and MacIntosh 2017). Importantly, the increased longitudinal bending stiffness will likely only be beneficial when achieved using a (downward) moderately stiff curved plate that is embedded within the midsole because this reduces negative MTP joint work without increasing the ankle moment, such as would occur if a flat, very stiff plate was placed directly below the insole (Stephen et al. 2025; Ortega et al. 2021; Rodrigo‐Carranza, Hoogkamer, et al. 2024; Beck et al. 2020). Similar to the mechanism of a higher longitudinal bending stiffness decreasing plantar flexion velocity, in some studies, more compliant (Petrella et al. 2026) and resilient (Ferris et al. 2026) midsoles also decrease ankle plantar flexion velocity. In turn, this could explain why some studies have observed reduced ankle plantar flexion velocity in AFT—that combines both elements—compared to control shoes (Matties et al. 2024).

### 3.3 Lower Leg Muscle Force Production to Accommodate Mechanical Demands

The “cost of cushioning” hypothesis suggests that external cushioning reduces the metabolic cost associated with leg muscular force production required to support body weight, with the metabolic cost benefit being up to ∼1.6% (Franz et al. 2012; Tung et al. 2014; Frederick et al. 1983). Because AFT is characterized by highly compliant midsole foams, the cushioning provided by these midsoles could be another contributor to the observed RE benefit (Worobets et al. 2014; Ferris et al. 2026; Petrella et al. 2026; Baumann et al. 2025), with other studies also indirectly supporting this (Joubert et al. 2024; Flores et al. 2019). For example, softer shoes (difference of 25 Asker C points) with similar energy return improved RE by ∼3% (Petrella et al. 2026). Such findings may be explained by softer shoes reducing the need for knee flexion (i.e., increasing leg stiffness (Needles et al. 2026; Petrella et al. 2026)), thereby potentially reducing knee joint moments and the associated metabolic cost of generating knee extensor force, despite a similar or even higher vertical peak ground reaction force (Hoogkamer et al. 2018; Petrella et al. 2026). Notably, while at first glance some findings appear to challenge the “cost of cushioning” hypothesis, as differences in midsole hardness did not affect RE (Rodrigo‐Carranza et al. 2026), a closer examination suggests that the relatively small difference in hardness between conditions (5 Asker C points) was likely insufficient to elicit meaningful changes in RE. Therefore, a relatively large difference in midsole hardness might be required before measurable benefits in RE emerge. Overall, whether compliant midsoles benefit RE therefore likely depends on both the stiffness of the underlying surface, with stiffer surfaces increasing the need for softer shoes, and on the relative difference between the AFT and control shoe.

The lower negative leg muscle work (i.e., eccentric muscle force production) associated with AFT may also reduce muscle damage. Indeed, running in AFT has been reported to result in less (calf) muscle soreness, lower markers of muscle damage and/or inflammation (e.g., Lactate dehydrogenase concentrations), and faster perceived recovery after intense or long workouts (Castellanos‐Salamanca et al. 2023; Lyons et al. 2025; Kirby et al. 2019), although this has not been observed in the quadriceps (Lee et al. 2026). Consequently, the reduced muscle damage and overall physiological strain associated with AFT use might enable athletes to tolerate a higher training volume and/or intensity (Kirby et al. 2019), thereby promoting greater cumulative training adaptations (Bishop et al. 2019; Molmen et al. 2025) and improvements in endurance performance (Zanini et al. 2026; Casado et al. 2021). In contrast to these assertions, preliminary findings suggest slightly *smaller* adaptations (e.g., by ∼0.5% for RE) after 8 weeks of training in AFT compared to control shoes (Matties et al. 2024). Although these findings should be interpreted with caution because of the very small sample size, they could hint at reduced adaptations if the improved RE with AFT is not used to increase training volume and/or intensity.

Further, AFT could also mitigate fatigue‐related declines in performance within a given session by preserving neuromuscular function and metabolic efficiency (partly as a result of reduced muscle damage). For example, although two studies report that RE deteriorated to a similar extent in AFT and control shoes during prolonged running (80 min (Madsen et al. 2025) or 90 min (Schwalm et al. 2025)) or with plantar flexor fatigue (Bidois et al. 2026), Steele et al. (2026) reported smaller increases in $\dot{V}O_{2}$ and indices of neuromuscular fatigue over 60 min of running in AFT compared with a control shoe. Similarly, Black and colleagues (Black et al. 2021) observed that the RE benefit with AFT compared with a control shoe was greater when running with the presence of muscle damage (∼4.6%) compared with the nondamaged condition (∼3.2%). Collectively, the observed performance benefits of AFT may therefore not only reflect their positive effects on RE measured under fresh conditions, but also their effects on attenuating exercise‐induced muscle damage and thereby enhancing physiological resilience and training practices.

### 3.4 Relative Importance of Different Shoe Properties for RE

Public perception often attributes the performance benefits of AFT to the carbon plate, as reflected in their common label as “carbon plated shoes”. A recent meta‐analysis, however, indicates that the mechanical effects associated with individual shoe properties, such as energy return and longitudinal bending stiffness, each have only small, statistically nonsignificant effects on RE, with improvements of approximately ∼1% when considered in isolation (Stephen et al. 2025). This suggests that no single property is sufficient to fully explain the performance benefits of AFT, pointing instead toward a synergistic effect of multiple features. Consequently, AFTs are best conceptualized as tools that redistribute mechanical work and load within the runner‐shoe system, rather than as devices that simply “return energy” (Burns and Joubert 2024; Kram 2022; Barrons et al. 2023). In line with a combined effect, one experimental study shows that both the longitudinal stiffening element and midsole foam independently contribute to improve RE, with estimated benefits of 1.8% and 2.2%, respectively (Ghanbari, Fletcher, et al. 2025). Notably, the sum of these effects reasonably approximates the ∼2.7% average meta‐analytical RE improvement reported across AFT shoes (Stephen et al. 2025), supporting the idea that additive contributions of multiple components underlie the overall benefit. Likewise, combining the isolated effects of midsole compliance, and resilience (energy return) yields a RE improvement (∼2.6%) that closely matches the meta‐analytical effect (Ferris et al. 2026). Importantly, midsole compliance was found to be more important than the energy return for the RE benefit in this study (Ferris et al. 2026).

However, the relative importance of each shoe property will inevitably vary between studies as a function of the reference shoe and surface, amongst other factors. When control shoes and/or the surface, for example, already possess high compliance, the relative advantage of compliant AFT might be reduced. Similarly, if the control shoes are relatively stiff, the benefit of additional longitudinal bending stiffness will be reduced. In this context, it is important to note that most treadmills have a relatively compliant surface (Colino et al. 2020). In line with previous suggestions (Kram 2022; Van Hooren et al. 2020), we therefore recommend the use of stiff treadmills in AFT research to best match the typical application of AFT on (stiff) road running surfaces.

### 3.5 Future Directions for Assessing the Performance Benefits of AFT

To mechanistically link shoe energy return with reductions in muscular force production (and improvements in running performance), methods and frameworks have been proposed to better capture the complex continuum of shoe loading (deformation) and unloading (rebound) both under benchtop conditions and in vivo. For benchtop testing (energy storage and return capacity), advanced material testing protocols have been developed that promisingly characterize the spatial material properties of midsoles, rather than assuming uniform properties across a region of the midsole (Mai et al. 2021). Although material testing protocols are valuable for benchmarking shoe energy return capacity across models, researchers and running shoe developers should recognize that energy‐return capacity does not necessarily translate linearly into in vivo performance. For example, benchtop testing typically uses slow loading rates (Scherrer et al. 2026), and treats the shoe as an isolated structure, despite the fact that during locomotion, the shoes experience fast loading rates, and the foot, shoe, and running surface together form an integrated, nonrigid mechanical system whose properties vary substantially across individuals, environments, and experimental contexts (Hofmann 2026). Underfoot testing methods such as “unified deformable segment analyses” have been proposed to estimate shoe deformation and rebound (mechanical work) in vivo (Matijevich et al. 2022), supporting the unique evaluation of both the timing and magnitude of shoe energy return (Matijevich et al. 2025). In vivo shoe deformation can also be estimated with extended motion capture marker sets that quantify midsole displacements, by analyzing in‐shoe plantar pressures to infer when, where, and for how long regions of the midsole are compressed (Matijevich et al. 2022), or with fast dual X‐ray videography methods. Key opportunities for future energy return research include the continued development of innovative methods for quantifying shoe and surface deformation alongside biological mechanical work, as well as designing shoe features that strategically manipulate both the magnitude and the timing of energy storage and return (e.g., hollow shoe structures (Liu et al. 2024), or stiffening plates that delay energy return (Ghanbari, Vienneau, et al. 2025)).

## 4 Individual Responses to AFT Usage

Despite robust improvements in RE observed at the group level, responses to AFT vary markedly between individuals. For example, when a single trial is conducted per shoe, the RE difference in AFT compared to traditional racing or conventional shoes ranges from a −14% impairment to a +13.3% benefit (Hebert‐Losier et al. 2022; Knopp et al. 2023; Muniz‐Pardos et al. 2025). However, this difference decreases by almost half to +/− ∼7% when multiple trials are averaged (Barnes and Kilding 2019; Kuzmeski et al. 2025; Hoogkamer et al. 2018; Hunter et al. 2019), indicating that almost 50% of this variability can be attributed to biological trial‐to‐trial variation and technological measurement error variability (Nielsen, Heyde, et al. 2022; Barrons et al. 2024; Beltrami et al. 2025; Van Hooren et al. 2026). Nevertheless, inter‐individual differences of ∼7% indicate that variability in how runners respond to AFT persists even when repeated testing is used to decrease variability (Barnes and Kilding 2019; Kuzmeski et al. 2025; Hoogkamer et al. 2018; Werkhausen et al. 2024; Hunter et al. 2019; Madsen et al. 2025; Matijevich et al. 2022). The robust effect of AFT shoes on RE at the group level, alongside a highly variable response at the individual level, suggests that the effect is not solely a direct mechanical effect resulting from specific shoe properties, as discussed in Section 3, but that it is mediated by individual biomechanical, anthropometric, perceptual, and/or contextual factors.

### 4.1 Running Biomechanical Mechanisms Underlying Individual Responses

Differences in running technique may partly explain the variability in RE benefit provided by AFT. For example, using unified deformable segment analyses, Matijevich et al. (2025) showed that runners who had the greatest increase in positive foot‐plus‐shoe work in AFT compared with a traditional shoe also had the greatest decrease in positive ankle work. Because reductions in positive ankle work may improve RE, runners who are better able to exploit shoe deformation—by increasing mechanically useful foot‐plus‐shoe work at appropriate phases of stance—could experience greater reductions in ankle work and, in turn, larger RE benefits (Burns and Joubert 2024; Kram 2022). Similarly, runners who exhibit larger decreases in their ankle plantar flexor velocity in response to the higher longitudinal bending stiffness in AFT could conceivably experience larger RE benefits. Finally, individuals whose running mechanics generate higher foot–ground reaction forces could have an increased requirement for cushioning to attenuate these forces. Consequently, such individuals might experience enhanced RE benefits when using highly compliant AFT, although the larger midsole compression may simultaneously enhance shoe energy return, possible partly/fully offsetting this benefit. However, all three of these proposed relationships remain to be directly tested experimentally.

Despite these possible associations, studies assessing whether changes in running technique parameters are related to the RE benefit in AFT typically observe no relationships (Perry et al. 2025; Van Hooren et al. 2025; Hoogkamer et al. 2018; Martinez et al. 2024; Beck et al. 2020; Chollet et al. 2023). For example, although increases in ground contact time as an indirect indicator of slower muscle shortening velocity demands could be hypothesized to be associated with the RE benefit in AFT, this effect is not supported by current evidence. These findings collectively suggest that the evaluation of spatiotemporal metrics does not sufficiently reflect muscle‐tendon‐level mechanics and the associated energetic cost.

### 4.2 Anthropometrical Mechanisms Underlying Individual Responses

Anthropometric characteristics may also moderate individual responses to AFT. Indeed, body mass, foot size, and lever arms influence the magnitude of midsole and plate deformation under load, thereby altering the effective mechanical behavior of the shoe–runner system (Burns and Joubert 2024; Kram 2022). Similarly, body mass may influence the degree of shoe cushioning required, and thereby the RE benefit from highly compliant AFT compared to traditional racing flats. However, studies assessing whether isolated anthropometrical variables influence the response to AFT have typically reported no relationships (Seglina et al. 2026; Van Hooren et al. 2025; Hebert‐Losier et al. 2026). For example, Van Hooren et al. (2025) found no correlation between body mass, Achilles tendon moment arms, or calf muscle architecture, and the AFT benefit across two different AFT shoes compared to a racing flat or conventional running shoe. One preliminary study, however, indicated that runners with greater MTP stiffness might benefit less from AFT (Hazelwood et al. 2025). Similarly, another study indicates that normalized gastrocnemius medialis passive stiffness and navicular drop correlate with the individual RE response (Hebert‐Losier et al. 2026). Although differences in MTP and gastrocnemius stiffness amongst others could therefore underlie part of the individual variations in RE response to AFT, these findings require confirmation across different AFT shoes and populations.

### 4.3 Familiarization, Habituation, and Comfort as Mechanisms Underlying Individual Responses

Familiarization, habituation and perceptual factors such as perceived comfort might also influence the response to AFT shoes, with individuals who are less familiarized or habituated to AFT, or perceive the shoes as less comfortable, possibly showing smaller RE benefits. In this context, familiarization refers to *short‐term* sensorimotor, cognitive, and biomechanical changes over brief exposure, whereas habituation refers to *long‐term* sensorimotor, cognitive, and biomechanical changes resulting from repeated exposure to a stimulus. However, evidence regarding the importance of the presence and magnitude of familiarization and habituation is equivocal. For example, Beltrami and colleagues (Beltrami et al. 2025) concluded that there could be a familiarization effect with AFT across four trials because the difference between two AFT shoes increased with more trials. In contrast, another study indicated no familiarization effect (Nielsen, Heyde, et al. 2022), although only two trials were used. Similarly, some studies report habituation effects (Matties et al. 2024; Matties and Rowley 2026), whereas others do not (Schwalm et al. 2024). These differences may at least partly reflect methodological aspects. For example, habituation effects (possibly increased benefit of AFT) could be masked by an interaction with shoe degradation due to repeated loading (decreased benefit). Nevertheless, given possible familiarization and habituation effects, future studies should consider these aspects when assessing factors that influence the individual response. Regardless of the possible impact on RE, habituation might also be relevant for other outcomes, including musculoskeletal adaptation to altered loading patterns and a potential reduction in overuse injury risk.

Perceptual factors such as comfort, perceived propulsion, and confidence also influence neuromuscular coordination and pacing behavior, thereby indirectly affecting RE and performance (Castellanos‐Salamanca et al. 2023; Hébert‐Losier, Knighton, et al. 2025). A greater perceived comfort of AFT relative to racing flats, conventional running shoes, or runners' own shoes was, however, not associated with a greater RE benefit (Van Hooren et al. 2025), suggesting that perceived comfort does not represent a primary determinant from which RE benefits can be inferred. In line with this, placebo effects do not appear to play a primary role in explaining the positive effects of AFT (Kram 2022; Hébert‐Losier, Pfister, et al. 2025).

### 4.4 Future Directions for Investigating Individual Responses

Collectively, the absence of clear univariate predictors suggests that individual responses likely emerge from the interaction of multiple factors rather than from any single characteristic. Consequently, understanding individual response profiles represents a key challenge for future AFT research. Future studies on this topic should consider the following aspects: first, most studies assessing RE across different shoes use a single running test per shoe. However, this is known to introduce substantial variability, therefore reducing the sensitivity to detect relationships between individual characteristics and the response to specific shoes (Figure 3). Thus, repeated trials and reliable equipment and measurements are recommended. Future studies should also consider the interaction between different individual characteristics instead of isolated variables, and factors not yet investigated (e.g., cognitive, psychological or cultural factors). Such approaches will therefore require multivariable statistical modeling or machine‐learning approaches that can account for interactions among multiple factors, instead of traditional univariate correlation analyses. With regard to biomechanical factors, research should explore running technique outcomes that better reflect muscle‐tendon level mechanics (e.g., joint angular velocities and moments). Fatigue represents an additional and often under‐considered moderator, with shoe–biomechanics interactions observed under fresh laboratory conditions possibly changing during prolonged or fatiguing running. Consequently, individual responses assessed during short, steady‐state trials might not fully reflect responses during races or high‐fatiguing training sessions.

**FIGURE 3:**
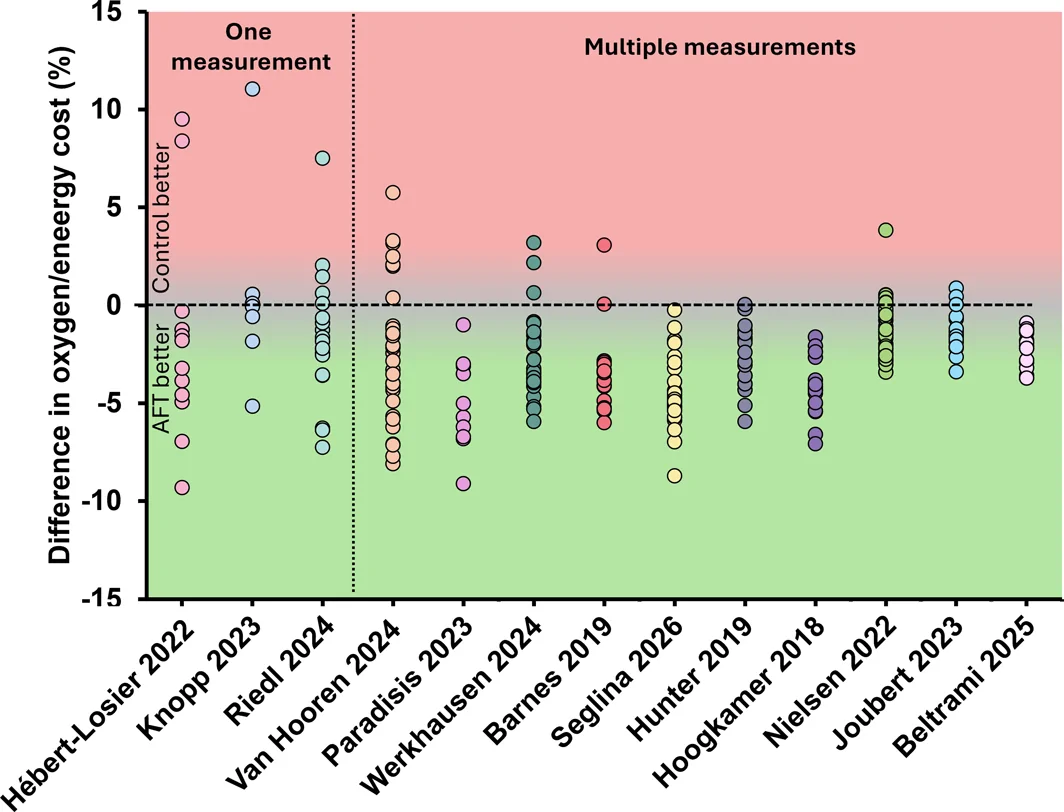
Individual response in RE to AFT use in studies from various labs doing one measurement per shoe (left of dashed line), or two or more measurements per shoe (right of dashed line). Studies are organized from having higher variability (left) to lower variability (right). Note the typical larger variability in response to AFT in studies doing one measurement compared to those doing multiple measurements. For example, Beltrami and colleagues (Beltrami et al. 2025) found no negative response when averaging across four trials, whereas negative responses were observed more frequently with fewer trials per shoe. Nevertheless, variability in the individual response remains also when averaging multiple measures. Individual data points were obtained from the authors, or extracted using WebPlot digitizer. For Hebert‐Losier et al. (2022) dots depict the energy cost of the Nike Vaporfly versus racing flat at 80% $\dot{V}O_{2}$ max. For Knopp et al. (2023), the comparison shows AFT shoe 1 versus the racing flat in well‐trained athletes. For Riedl et al. (2024), the figure shows the Hoka AFT versus the participant's own shoes at the first speed. For Van Hooren et al. (2025) dots show the Decathlon AFT versus Decathlon control shoe. For Paradisis et al. (2023), the comparison between the AFT and control shoe is shown at 80% *v* $\dot{V}O_{2}$ max. For Barnes and Kilding (2019), data points depict the difference between the Adidas and Vaporfly at 16 km h^−1^. For Seglina et al. (2026), points reflect the average across the different speeds. For Hunter et al. (2019), we show a comparison between the Nike Vaporfly and Adidas Boost. For Hoogkamer et al. (2018), the datapoints depict the Nike Vaporfly versus the control shoe at 18 km h^−1^. For Nielsen, Heyde, et al. (2022) we used the AFT versus Adidas control shoe on average over two measurements on day 1. For Joubert et al. (2023), the comparison shows the highly cushioned AFT versus the control shoe at 12 km h^−1^. Finally, for Beltrami et al. (2025), we used the treadmill condition with four measurements per shoe.

## 5 Effects of AFT Usage on Running Injury Occurrence

Running performance shows strong associations with training characteristics such as running volume (Zanini et al. 2026; Casado et al. 2021). Therefore, the ability to follow a training or race program without interruption due to injury is of major importance not only for performance but also for short‐ and long‐term health. Although the role played by shoes in the development of running injury has been debated extensively over the last few decades (Malisoux and Theisen 2020), there is consensus that shoes can alter both loading magnitude and load distribution across the lower limbs, thereby indirectly influencing injury risk. In other words, shoes in themselves do not cause injuries, but act as an effect‐modifier that interacts with training load by altering running biomechanics.

Direct evidence relating AFT use to injury risk remains sparse, likely due to methodological challenges inherent in randomized trials on injury risk, and the lack of large injury databases stratified by shoe type and injury site allowing to address causal questions and explicitly apply causal‐inference design and analysis methods. As a result, current evidence is insufficient to determine whether AFT confers a protective effect or increases injury risk. For example, while four prospective studies have investigated AFT use and injury risk, they are typically characterized by a very high risk of bias, such as lack of a control group and a small number of injuries (*n* = 8) (Bonato et al. 2024), no randomization and adjustment for possible confounding factors (Brüggemann et al. 2025; Arndt et al. 2025), and no differentiation between injury risk in maximalist and AFT shoes (Bruggemann et al. 2025). In the only randomized controlled trial performed to date (published only as an abstract), 195 runners randomized to wear AFTs had fewer injuries and less reported pain when undertaking a 12‐week half‐marathon training program compared with runners wearing conventional running shoes (Ryan and Farina 2025).

Overall, the current evidence does not allow for robust conclusions regarding the impact of AFT on injury risk, and high‐quality research explicitly applying causal‐inference design is therefore needed to investigate the relationship between AFT and injuries. Although randomized controlled trials are considered the gold standard for addressing causality, applying such a design is difficult because runners are unlikely to agree to wear AFT or control shoes exclusively for all their running sessions over several months. An intervention based on partial use of AFT shifts the focus to the alternation of shoes strategy, where use of multiple shoes in a training period has been associated with reduced risk of running‐related injury (Malisoux et al. 2015). Furthermore, the transition to AFT use should be considered. Nevertheless, some research questions could also be addressed using observational study designs, where transparent reporting of a clear causal framework, and careful consideration of possible bias with appropriate design (e.g., participants inclusion) and analysis (e.g., adjustment), could allow the results to be interpreted causally (Hernán and Robins 2020). In any case, future research questions should assess for which runners AFTs may be beneficial or detrimental, and under which conditions (e.g., training regimen, running technique, adaptation period) injury rates differ.

### 5.1 Effect of AFT on Injury‐Related Biomechanics

Because most injuries in distance running are overuse injuries and result from repetitive loading of the musculoskeletal system (Bertelsen et al. 2017; Edwards 2018), understanding how AFT alters biomechanical loading at common sites of injury indirectly informs our understanding of injury risk. However, only a few studies have assessed internal biomechanical loading at common injury sites in AFT. Werkhausen et al. (2024) observed reduced cumulative tibial damage per km of running in AFT compared with a control shoe. Another study also reported lower ankle contact forces in AFT versus non‐AFT control shoes (Kim and Ahn 2025), which might reflect lower tibial injury risk (because the tibia bears ∼90% of the ankle contact force). These studies therefore suggest lower tibial injury risk when running in AFT, although this was observed when running at the same speed in each shoe type, whereas individuals may run at a faster speed in AFTs in practice, which in turn increases tibial loading (Van Hooren et al. 2024). Another preliminary study indicated lower axial metatarsal stresses, but higher bending moments in AFT (Milner et al. 2025). Because bending moments contribute substantially to the normal stress experienced by the metatarsals, this could reflect increased metatarsal injury risk with AFT. Although one study reported no difference in the peak ankle or knee joint moments between AFT and non‐AFT shoes (Werkhausen et al. 2024), three other studies report lower peak ankle joint moments in AFT versus non‐AFT shoes (Martinez et al. 2024; Hoogkamer et al. 2019; Bruneau et al. 2026), hinting at a lower Achilles tendon injury risk. Collectively, these findings therefore could reflect a reduced injury risk at some sites (tibia, Achilles tendon) but an increase at others (metatarsals and tarsal) (Figure 4). However, whether altered loading leads to increased or decreased injury risk depends on the tissue‐specific load capacity and remodeling ability. Further, it remains unknown whether the reduced musculoskeletal loading at some sites induces a “detraining” effect with chronic exposure that may subsequently increase injury risk.

**FIGURE 4:**
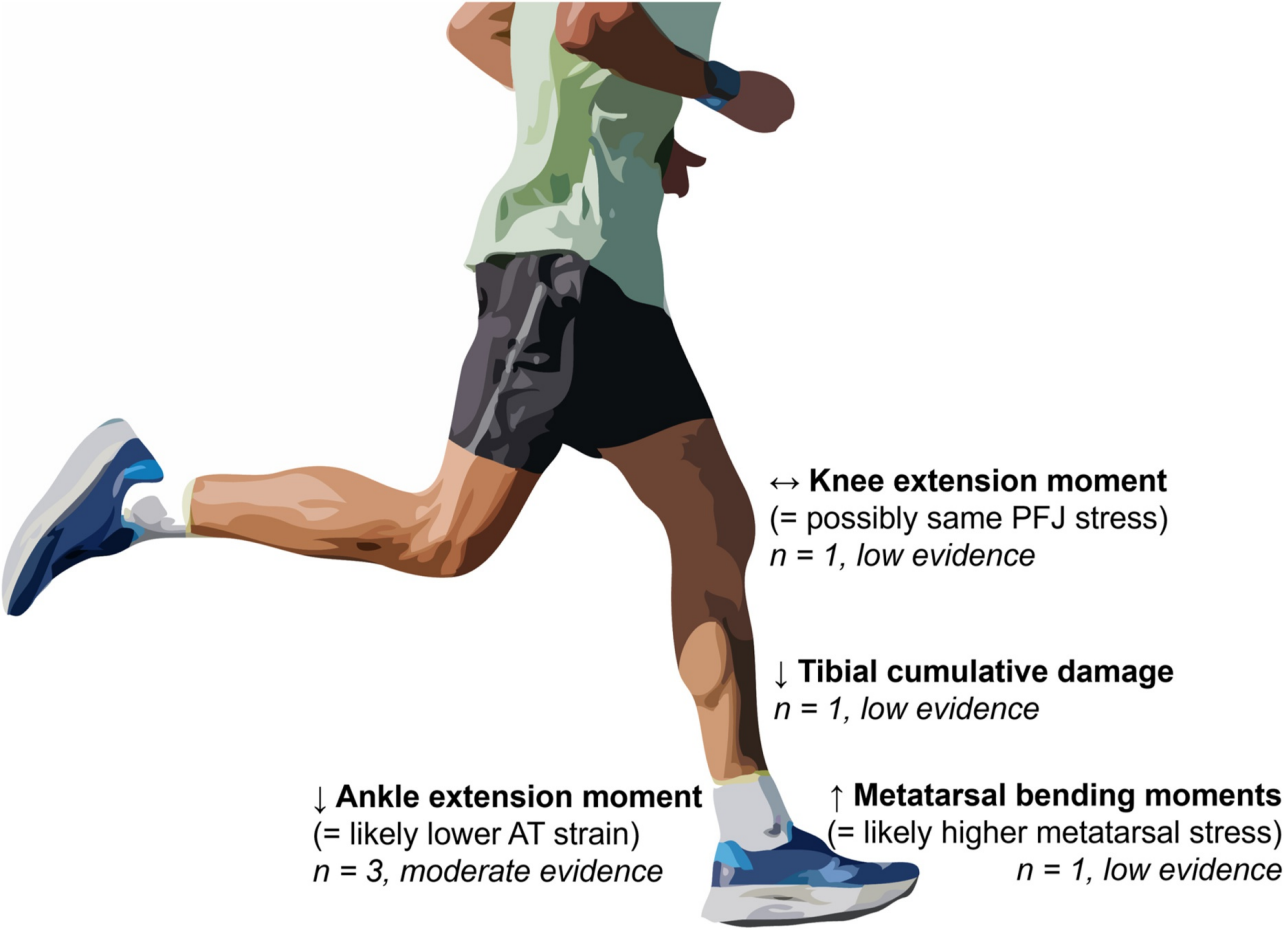
Changes in biomechanical loading with AFT versus non‐AFT when running at equivalent speed. Note that altered loading does not necessarily correspond to altered injury risk because this also depends on the tissue‐specific load capacity and remodeling ability. AT = Achilles tendon; PFJ = patellofemoral joint.

Other experimental studies have assessed isolated components present within AFT in relation to biomechanical loading. These studies showed that compared with flat plates, curved carbon plate designs can reduce sagittal‐plane hip and knee joint angles and hip flexion moments, while increasing tibialis anterior activation patterns (Xu et al. 2025). Carbon plate thickness and geometry have also been shown to influence the distribution of metatarsal stresses (Guan et al. 2025; Song et al. 2023). For example, flat carbon plates placed at the level of the outsole decreased metatarsal stress when compared with no plate, whereas curved plates in the midsole—as present in most AFT shoes—increased metatarsal stress at the second metatarsal (Song et al. 2023), which is (along with the third metatarsal) the most common site of metatarsal injuries. A subsequent study showed a similar trend for an increase in stress at the second and third metatarsals—but decreases at all other metatarsals—with curved plates of different stiffness compared with no plate (Song et al. 2024). Further, a greater toe spring (upward curvature of the shoe sole at the forefoot) has been shown to reduce metatarsal and hallux stresses (Li et al. 2026). Carbon plate stiffness has also been shown to influence plantar fascia stress, with stiffer plates lowering plantar fascia stress near the calcaneus (Qian et al. 2026). Importantly, all these studies used data from only one runner, typically at only one running speed, and further research is therefore required to confidently generalize these findings. Another preliminary study showed no difference in cumulative Achilles tendon weighted impulse when a carbon plate was inserted below the insole compared with no carbon plate (Firminger et al. 2019), whereas another study showed an increase in the peak Achilles tendon force and impulse with stiffer curved longitudinal plates (Lin et al. 2026). Finally, midsole compliance in AFT could also impact injury risk. For example, a study on 800+ runners showed that softer (non‐AFT) shoes were associated with reduced injury risk (Malisoux, Delattre, et al. 2020). This might be because more compliant midsoles reduce contact forces at lower limb joints (Meardon et al. 2018), attenuate impact peak force of the high frequency signal (Malisoux et al. 2015), and attenuate high‐frequency vibration signals (Hernán and Robins 2020; Malisoux et al. 2021; Trama et al. 2023), which in turn could decrease tissue damage.

### 5.2 Future Directions for Investigating Injury Risk and Injury‐Associated Biomechanics

Overall, although there is evidence that AFT alters lower limb running mechanics, it is presently unclear whether this increases or decreases the risk of injury at any given site and tissue, or overall injury risk. Furthermore, AFT might allow individuals to run faster or increase their total training load, which in itself influences injury risk (both negatively due to musculoskeletal damage, and positively due to tissue adaptation). Nevertheless, given the reported alterations in biomechanical loading with AFT, some consideration needs to be given to their incorporation in return‐to‐running programs after injury or when introducing them into training programs for the first time. For example, lower tibial loading may mean that AFT shoes could be advantageous during rehabilitation from tibial bone stress injuries by offloading symptomatic tissues while maintaining running exposure, assuming that running speed is not increased. Conversely, the possible higher metatarsal loading could warrant a delayed incorporation of AFT during return‐to‐running programs after metatarsal injuries.

Regarding future directions, we encourage high‐quality randomized controlled trials or observational studies carefully designed to address causal questions to assess the effect of AFT use on injury risk. We also encourage more studies into specific internal biomechanical loading (as opposed to external biomechanics, which are not always associated with internal loading (Matijevich et al. 2019; Scott and Winter 1990; Zandbergen et al. 2025)) as well as studies that quantify changes in tissue properties over time as an indicator of tissue health (e.g., HR‐pQCT for tibial bone health or ultrasound for tendon health). Similar to the individual response to AFT with regard to RE, individual responses have been shown for injury‐related biomechanical outcomes such as joint contact forces in different shoes (Meardon et al. 2018). Therefore, research is also needed to assess individual responses in the context of running injury risk in AFT.

Finally, most studies assessing the effects of AFT on biomechanics (and RE) have used relatively new shoes. However, it is known that repeated loading of running shoes changes their mechanical properties, leading to reduced energy absorption (Allen et al. 2020; Aimar et al. 2024) and higher energy loss (Rodrigo‐Carranza, Hoogkamer, et al. 2024), which could attenuate the beneficial effect of shoes on RE and increase injury risk. Importantly, previous work has shown that compliant (PEBA) midsole foams often used in AFT degrade more rapidly than stiffer (EVA) midsole foams (Aimar et al. 2024; Lippa et al. 2017). For example, Rodrigo‐Carranza, et al. (Rodrigo‐Carranza, Hoogkamer, et al. 2024) showed that AFT with PEBA foam degrades faster than shoes with EVA foam, as evidenced by greater worsening of RE and larger changes observed during material testing. However, this study assessed these properties only after 450 km of running, and with only one degraded shoe, which was subsequently used among multiple runners in the laboratory. Further research is therefore required to study the time course of degradation, and also its effect on injury risk. Because of the faster degradation of AFT, it is also important that the accumulated distance in the tested running shoes is reported, because this is only rarely done (e.g. (Xiao et al. 2026; Joubert et al. 2024; Schwalm et al. 2024)).

## 6 Conclusion

Current evidence indicates that AFT confers meaningful improvements in running economy and performance, with metabolic savings of approximately 2%–4% on average and performance gains of 1%–3%. These benefits arise from a combination of acute effects and potentially longer‐term adaptations. Proposed acute mechanisms include reduced muscle force production through enhanced mechanical energy return, reduced metabolic cost associated with accommodating the mechanical running demands due to compliant foams, and greater efficiency of producing force associated with increased longitudinal bending stiffness and possibly the curved shoe geometry. The relative contribution of these mechanisms likely depends on contextual factors, such as running surface, running speed and individual responses. Additionally, emerging findings suggest that AFT may reduce muscle damage/inflammation and soreness, with possible implications for improved physiological resilience, as well as runners' training volume, frequency and intensity. From a biomechanical perspective, AFT use appears to redistribute loading, potentially increasing stress at the metatarsals and tarsal while reducing loading at the tibia and possibly the Achilles tendon. However, the implications for injury risk remain inconclusive due to limited high‐quality evidence. Importantly, substantial inter‐individual variability in response to AFT has been consistently observed, which could influence both running performance benefits and injury risk. The factors underpinning this variability remain poorly understood and represent a key area for future research.

## Conflicts of Interest

Some authors have received funding for contracted work related to AFT.

## Data Availability

Data sharing not applicable to this article as no datasets were generated or analyzed during the current study.

## References

[ejsc70266-bib-0001] 3rd Martinez, E. , W. Hoogkamer , D. W. Powell , and M. R. Paquette . 2024. “The Influence of “Super‐Shoes” and Foot Strike Pattern on Metabolic Cost and Joint Mechanics in Competitive Female Runners.” Medicine & Science in Sports & Exercise 56, no. 7: 1337–1344. 10.1249/MSS.0000000000003411. 38376997

[ejsc70266-bib-0002] Agresta, C. , C. Giacomazzi , M. Harrast , and J. Zendler . 2022. “Running Injury Paradigms and Their Influence on Footwear Design Features and Runner Assessment Methods: A Focused Review to Advance Evidence‐Based Practice for Running Medicine Clinicians.” Frontiers in Sports and Active Living 4: 815675. 10.3389/fspor.2022.815675. PMC8959543 35356094

[ejsc70266-bib-0003] Aimar, C. , L. Orgéas , R. S. Rolland du , L. Bailly , and D. Ferré Sentis . 2024. “Compression Fatigue of Elastomeric Foams Used in Midsoles of Running Shoes.” Footwear Science 16, no. 2: 93–103. 10.1080/19424280.2024.2317881.

[ejsc70266-bib-0004] Allen, T. , M. Pagan , R. Martin , and O. Duncan . 2020. “Effect of Rest Periods on Mechanical Ageing of Running Shoes.” Proceedings 49, no. 1: 138. 10.3390/proceedings2020049138.

[ejsc70266-bib-0005] Arndt, T. , Ö Dahlström , J. Jacobsson , T. Timpka , and J. Kvist . 2025. “Longitudinal Follow‐Up of Running Injury Incidence in Recreational Runners in Relationship to the Use of Advanced Footwear Technology Shoes.” Supplement, Footwear Science 17, no. S1: S219–S220. 10.1080/19424280.2025.2490694.

[ejsc70266-bib-0006] Askew, G. N. , S. Hill , M. Payne , T. D. Stewart , D. Charles , and A. P. Brown . 2026. “Running Economy Benefits of Shoes Incorporating Advanced Footwear Technology Decrease With Increasing Incline and Are Negligible Above Moderate Gradients.” Scandinavian Journal of Medicine & Science in Sports 36, no. 5: e70298. 10.1111/sms.70298. PMC13168684 42121353

[ejsc70266-bib-0007] Barnes, K. R. , and A. E. Kilding . 2019. “A Randomized Crossover Study Investigating the Running Economy of Highly‐Trained Male and Female Distance Runners in Marathon Racing Shoes versus Track Spikes.” Sports Medicine 49, no. 2: 331–342. 10.1007/s40279-018-1012-3. 30374945

[ejsc70266-bib-0008] Barrons, Z. B. , V. Rodrigo‐Carranza , M. Bertschy , and W. Hoogkamer . 2024. “The Fallacy of Single Trials: The Need for Multiple Trials in Assessing Running Economy Responses in Advanced Footwear Technology.” Sports Medicine 54, no. 6: 1357–1360. 10.1007/s40279-023-01991-1. 38407747

[ejsc70266-bib-0009] Barrons, Z. B. , J. W. Wannop , and D. J. Stefanyshyn . 2023. “The Influence of Footwear Midsole Thickness on Running Biomechanics and Running Economy in Female and Male Runners.” Footwear Science 15, no. 3: 155–160. 10.1080/19424280.2023.2218321.

[ejsc70266-bib-0010] Baumann, G. A. , K. Biedermann , E. Item , C. M. Spengler , and F. G. Beltrami . 2025. “The Effect of Midsole Thickness on Running Economy, Spatiotemporal Values and Perceptions of Comfort and Exertion in Well‐trained Runners: A Randomized, Cross‐Over Trial.” Sports Medicine‐Open 11, no. 1: 108. 10.1186/s40798-025-00911-z. PMC12488538 41032180

[ejsc70266-bib-0011] Beck, O. N. , P. R. Golyski , and G. S. Sawicki . 2020. “Adding Carbon Fiber to Shoe Soles May Not Improve Running Economy: A Muscle‐Level Explanation.” Scientific Reports 10, no. 1: 17154. 10.1038/s41598-020-74097-7. PMC7555508 33051532

[ejsc70266-bib-0012] Beltrami, F. G. , G. A. Baumann , K. Biedermann , and C. M. Spengler . 2025. “On the Fallacy of Single Trials: A Proposal to Reduce Variability in Running Shoe Research.” Sports Medicine 55, no. 4: 1051–1055. 10.1007/s40279-025-02180-y. 40063188

[ejsc70266-bib-0013] Bermon, S. , F. Garrandes , A. Szabo , I. Berkovics , and P. E. Adami . 2021. “Effect of Advanced Shoe Technology on the Evolution of Road Race Times in Male and Female Elite Runners.” Frontiers in Sports and Active Living 3: 653173. 10.3389/fspor.2021.653173. PMC8100054 33969296

[ejsc70266-bib-0014] Bertelsen, M. L. , A. Hulme , J. Petersen , et al. 2017. “A Framework for the Etiology of Running‐Related Injuries.” Scandinavian Journal of Medicine & Science in Sports 27, no. 11: 1170–1180. 10.1111/sms.12883. 28329441

[ejsc70266-bib-0015] Bertschy, M. , V. Rodrigo‐Carranza , E. W. C. Wilkie , et al. 2025. “Self‐Perceived Middle‐Distance Race Pace is Faster in Advanced Footwear Technology Spikes.” Journal of Sport and Health Science 14: 100975. 10.1016/j.jshs.2024.100975. PMC11863278 39222878

[ejsc70266-bib-0016] Bidois, B. , C. Cumming , M. Giandolini , A. P. Nguyen , and K. Hébert‐Losier . 2026. “Economy Benefits of Running in Advanced Footwear Technology Shoes Remain With Plantarflexion Fatigue.” European Journal of Applied Physiology 126, no. 7: 1–15. 10.1007/s00421-026-06190-0. PMC13380573 41831056

[ejsc70266-bib-0017] Biewener, A. A. , and M. A. Daley . 2007. “Unsteady Locomotion: Integrating Muscle Function With Whole Body Dynamics and Neuromuscular Control.” Journal of Experimental Biology 210, no. Pt 17: 2949–2960. 10.1242/jeb.005801. PMC2651961 17704070

[ejsc70266-bib-0018] Bishop, D. J. , J. Botella , and C. Granata . 2019. “CrossTalk Opposing View: Exercise Training Volume is More Important Than Training Intensity to Promote Increases in Mitochondrial Content.” Journal of Physiology 597, no. 16: 4115–4118. 10.1113/JP277634. 31309570

[ejsc70266-bib-0019] Black, M. I. , S. H. Kranen , S. Kadach , et al. 2021. “Highly Cushioned Shoes Improve Running Performance in Both the Absence and Presence of Muscle Damage.” Medicine & Science in Sports & Exercise 54, no. 4: 633–645. 10.1249/MSS.0000000000002832. PMC8920011 34816812

[ejsc70266-bib-0020] Bolliger, A. , C. M. Spengler , and F. G. Beltrami . 2026. “Impact of Advanced Footwear Technology on Running Economy at Slower Running Speeds: A Randomised, Cross‐Over Investigation.” Sports Medicine‐Open 12, no. 1: 12. 10.1186/s40798-026-00977-3. PMC12913831 41703164

[ejsc70266-bib-0021] Bonato, M. , F. Marmondi , E. L. Faelli , C. Pedrinelli , L. Ferraris , and L. Filipas . 2024. “Advanced Footwear Technology in Non‐Elite Runners: A Survey of Training Practices and Reported Outcomes.” Sports 12, no. 12: 356. 10.3390/sports12120356. PMC11679133 39728896

[ejsc70266-bib-0022] Bruggemann, G. P. , T. Esser , S. Rehorst , et al. 2025. “The Influence of Different Running Shoe Technologies on the Musculoskeletal Load in Running and Injury Outcomes in Recreational Runners.” Sportverletzung‐Sportschaden 39, no. 2: 77–86. 10.1055/a-2563-4935. 40398446

[ejsc70266-bib-0023] Brüggemann, G.‐P. , E. Hirschhaeuser , and S. Rehorst . 2025. “How Conventional or Technologically Advanced Running Shoes Influence the Risk and Location of Injuries: A Prospective Interventional Study of 414 Amateur Runners.” SportRχiv, Preprint.

[ejsc70266-bib-0024] Bruneau, M. M. , L. W. Gaudette , E. Sirls , et al. 2026. “Biomechanics Associated With Bone Stress Injuries While Using Advanced Footwear Technology in Elite Distance Runners.” Supplement, Pharmacy Management R 18, no. S2: S143–S150. 10.1002/pmrj.70153. 42026723

[ejsc70266-bib-0025] Burns, G. T. , and D. P. Joubert . 2024. “Running Shoes of the Postmodern Footwear Era: A Narrative Overview of Advanced Footwear Technology.” International Journal of Sports Physiology and Performance 19, no. 10: 975–986. 10.1123/ijspp.2023-0446. 39117307

[ejsc70266-bib-0026] Burns, G. T. , and N. Tam . 2020. “Is it the Shoes? A Simple Proposal for Regulating Footwear in Road Running.” British Journal of Sports Medicine 54, no. 8: 439–440. 10.1136/bjsports-2018-100480. 31630088

[ejsc70266-bib-0027] Casado, A. , B. Hanley , J. Santos‐Concejero , and L. M. Ruiz‐Perez . 2021. “World‐Class Long‐Distance Running Performances Are Best Predicted by Volume of Easy Runs and Deliberate Practice of Short‐Interval and Tempo Runs.” Journal of Strength & Conditioning Research 35, no. 9: 2525–2531. 10.1519/JSC.0000000000003176. 31045681

[ejsc70266-bib-0028] Castellanos‐Salamanca, M. , V. Rodrigo‐Carranza , S. Rodríguez‐Barbero , J. M. González‐Ravé , J. Santos‐Concejero , and F. González‐Mohíno . 2023. “Effects of the Nike ZoomX Vaporfly Next% 2 Shoe on Long‐Interval Training Performance, Kinematics, Neuromuscular Parameters, Running Power and Fatigue.” European Journal of Sport Science 23, no. 7: 1315–1323. 10.1080/17461391.2023.2171907. 36680410

[ejsc70266-bib-0029] Chollet, M. , S. Michelet , N. Horvais , S. Pavailler , and M. Giandolini . 2023. “Individual Physiological Responses to Changes in Shoe Bending Stiffness: A Cluster Analysis Study on 96 Runners.” European Journal of Applied Physiology 123, no. 1: 169–177. 10.1007/s00421-022-05060-9. 36229743

[ejsc70266-bib-0030] Cigoja, S. , J. R. Fletcher , M. Esposito , D. J. Stefanyshyn , and B. M. Nigg . 2021. “Increasing the Midsole Bending Stiffness of Shoes Alters Gastrocnemius Medialis Muscle Function During Running.” Scientific Reports 11, no. 1: 749. 10.1038/s41598-020-80791-3. PMC7804138 33436965

[ejsc70266-bib-0031] Cigoja, S. , J. R. Fletcher , and B. M. Nigg . 2022. “Can Changes in Midsole Bending Stiffness of Shoes Affect the Onset of Joint Work Redistribution During a Prolonged Run?” Journal of Sport and Health Science 11, no. 3: 293–302. 10.1016/j.jshs.2020.12.007. PMC9189708 33359799

[ejsc70266-bib-0032] Colino, E. , J. L. Felipe , B. Van Hooren , et al. 2020. “Mechanical Properties of Treadmill Surfaces Compared to Other Overground Sport Surfaces.” Sensors‐Basel 20, no. 14: 3822. 10.3390/s20143822. PMC7412192 32659884

[ejsc70266-bib-0033] Corbí‐Santamaría, P. , M. Gil‐Calvo , A. Herrero‐Molleda , J. García‐López , D. Boullosa , and J. V. García‐Tormo . 2025. “The Impact of Advanced Footwear Technology on the Performance and Running Biomechanics of Mountain Runners.” Applied Sciences 15, no. 2: 531. 10.3390/app15020531.

[ejsc70266-bib-0034] Denis, Y. , H. Knoke , L. Braun , and S. Willwacher . 2024. “New Year, New Gear: Does the next Version of an Advanced Footwear Model Lead to Better Performance?” SportRχiv. 10.51224/SRXIV.482.

[ejsc70266-bib-0035] Edwards, W. B. 2018. “Modeling Overuse Injuries in Sport as a Mechanical Fatigue Phenomenon.” Exercise and Sport Sciences Reviews 46, no. 4: 224–231. 10.1249/JES.0000000000000163. 30001271

[ejsc70266-bib-0036] Ferris, J. , B. Hazelwood , S. N. Cheuvront , and J. S. Gottschall . 2026. “Midsole Foam Compliance is a Greater Contributor to Running Economy, Biomechanics, and Perception than Foam Resilience or Stack Height in Advanced Footwear Technology in Male Runners.” Footwear Science 18, no. 2: 121–131. 10.1080/19424280.2026.2642702.

[ejsc70266-bib-0037] Firminger, C. , S. Cigoja , M. J. Asmussen , J. R. Fletcher , B. Nigg , and B. Edwards . 2019. “Effect of Longitudinal Bending Stiffness and Running Speed on a Probabilistic Achilles Tendinopathy Model.” Supplement, Footwear Science 11, no. S1: S66–S68. 10.1080/19424280.2019.1606081.

[ejsc70266-bib-0038] Fletcher, J. R. , and B. R. MacIntosh . 2017. “Running Economy From a Muscle Energetics Perspective.” Frontiers in Physiology 8, no. 433: 433. 10.3389/fphys.2017.00433. PMC5479897 28690549

[ejsc70266-bib-0039] Flores, N. , N. Delattre , E. Berton , and G. Rao . 2019. “Does an Increase in Energy Return and/or Longitudinal Bending Stiffness Shoe Features Reduce the Energetic Cost of Running?” European Journal of Applied Physiology 119, no. 2: 429–439. 10.1007/s00421-018-4038-1. 30470873

[ejsc70266-bib-0040] Fohrmann, D. , M. Schaffarczyk , C. Menge , et al. 2026. “Biomechanical Factors Associated With Intraindividual Differences in Running Economy Across Advanced Footwear Technology Models in Long‐Distance Runners.” Sports Medicine‐Open 12, no. 1: 34. 10.1186/s40798-026-01005-0. PMC13004771 41860638

[ejsc70266-bib-0041] Franz, J. R. , C. M. Wierzbinski , and R. Kram . 2012. “Metabolic Cost of Running Barefoot Versus Shod: Is Lighter Better?” Medicine & Science in Sports & Exercise 44, no. 8: 1519–1525. 10.1249/MSS.0b013e3182514a88. 22367745

[ejsc70266-bib-0042] Frederick, E. 2022. “Let’s Just Call it Advanced Footwear Technology (AFT).” Footwear Science 14, no. 3: 131. 10.1080/19424280.2022.2127526.

[ejsc70266-bib-0043] Frederick, E. , T. Clarke , J. Larsen , and L. Cooper . 1983. “The Effects of Shoe Cushioning on the Oxygen Demands of Running.” In Biomechanical Measurement of Running Shoe Cushioning Properties Biomechanical Aspects of Sport Shoes and Playing Surfaces, Vol. 107. 114. University of Calgary.

[ejsc70266-bib-0044] Ghanbari, A. , J. R. Fletcher , A. Bradshaw , and B. M. Nigg . 2025. “Effects of the Curved Carbon Fibre Plate and PEBA Foam on the Energy Cost of Running and Muscle Activation.” Footwear Science 17, no. 2: 107–116. 10.1080/19424280.2025.2471420.

[ejsc70266-bib-0045] Ghanbari, A. , J. Vienneau , S. R. Nigg , and B. M. Nigg . 2025. “Effects of Selected Features of Advanced Footwear Technology on Lower Limb Joint Work.” Sports Biomechanics 24, no. 9: 2707–2721. 10.1080/14763141.2025.2508244. 40420560

[ejsc70266-bib-0046] Guan, L. , Y. Guo , H. Wang , Y. Wu , Y. Jia , and X. Zhu . 2025. “Effects of Carbon‐Fiber Plate Design on Foot Stress Injury Risk: A Finite Element Analysis.” Frontiers in Bioengineering and Biotechnology 13: 1723984. 10.3389/fbioe.2025.1723984. PMC12756066 41487954

[ejsc70266-bib-0047] Hata, K. , Y. Hamamura , H. Noro , et al. 2024. “Plantar Flexor Muscle Activity and Fascicle Behavior in Gastrocnemius Medialis During Running in Highly Cushioned Shoes With Carbon‐Fiber Plates.” Journal of Applied Biomechanics 40, no. 3: 192–200. 10.1123/jab.2023-0170. 38458184

[ejsc70266-bib-0048] Hazelwood, B. , W. Hoogkamer , D. W. Powell , and M. R. Paquette . 2025. “The Influence of Metatarsophalangeal Joint Stiffness on the Economy Savings Response From ‘Super‐Shoes’ in Female Runners.” Supplement, Footwear Science 17, no. S1: S13–S14. 10.1080/19424280.2025.2489682.

[ejsc70266-bib-0049] Healey, L. A. , and W. Hoogkamer . 2022. “Longitudinal Bending Stiffness Does Not Affect Running Economy in Nike Vaporfly Shoes.” Journal of Sport and Health Science 11, no. 3: 285–292. 10.1016/j.jshs.2021.07.002. PMC9189697 34280602

[ejsc70266-bib-0050] Hebert‐Losier, K. , C. Cumming , B. Bidois , A. P. Nguyen , and M. Giandolini . 2026. “Exploring Factors to Explain Interindividual Responses to Running Economy in Advanced Footwear Technology Shoes: A Randomised Crossover Laboratory Trial With an Explanatory Predictor Analysis.” Sports Medicine‐Open 12, no. 1: 117. 10.1186/s40798-026-01091-0. PMC13481640 42606775

[ejsc70266-bib-0051] Hebert‐Losier, K. , S. J. Finlayson , M. W. Driller , B. Dubois , J.‐F. Esculier , and C. M. Beaven . 2022. “Metabolic and Performance Responses of Male Runners Wearing 3 Types of Footwear: Nike Vaporfly 4%, Saucony Endorphin Racing Flats, and Their Own Shoes.” Journal of Sport and Health Science 11, no. 3: 275–284. 10.1016/j.jshs.2020.11.012. PMC9189709 33264686

[ejsc70266-bib-0052] Hébert‐Losier, K. , H. Knighton , S. Finlayson , and B. Peterson . 2025. “How Does It Feel to Run in Minimalist and Advanced Footwear Technology Shoes: A Qualitative Study Involving Male Recreational Runners.” PLoS One 20, no. 12: e0338743. 10.1371/journal.pone.0338743. PMC12742779 41452817

[ejsc70266-bib-0053] Hebert‐Losier, K. , and M. Pamment . 2023. “Advancements in Running Shoe Technology and Their Effects on Running Economy and Performance—A Current Concepts Overview.” Sports Biomechanics 22, no. 3: 335–350. 10.1080/14763141.2022.2110512. 35993160

[ejsc70266-bib-0054] Hébert‐Losier, K. , A. Pfister , S. J. Finlayson , J.‐F. Esculier , P. Lamb , and C. M. Beaven .2025. “Are Super Shoes a Super Placebo? A Randomised Crossover Trial in Female Recreational Runners.” Footwear Science 17, no. 2: 79–88. 10.1080/19424280.2025.2458330.

[ejsc70266-bib-0055] Hernán, M. A. , and J. M. Robins . 2020. Causal Inference: What if. Chapman & Hall.

[ejsc70266-bib-0056] Heyde, C. , A. Nielsen , K. Roecker , et al. 2022. “The Percentage of Recreational Runners That Might Benefit From New Running Shoes. A Likely Scenario.” Footwear Science 14, no. 3: 163–172. 10.1080/19424280.2022.2095042.

[ejsc70266-bib-0057] Hofmann, C. 2026. “The Increase in Energy Return for Advanced Footwear Technologies over Traditional Footwear May Be Non‐Trivial.” Footwear Science 18, no. 2: 99–106. 10.1080/19424280.2026.2637936.

[ejsc70266-bib-0058] Hoogkamer, W. , S. Kipp , J. H. Frank , E. M. Farina , G. Luo , and R. Kram . 2018. “A Comparison of the Energetic Cost of Running in Marathon Racing Shoes.” Sports Medicine 48, no. 4: 1009–1019. 10.1007/s40279-017-0811-2. PMC5856879 29143929

[ejsc70266-bib-0059] Hoogkamer, W. , S. Kipp , and R. Kram . 2019. “The Biomechanics of Competitive Male Runners in Three Marathon Racing Shoes: A Randomized Crossover Study.” Sports Medicine 49, no. 1: 133–143. 10.1007/s40279-018-1024-z. 30460454

[ejsc70266-bib-0060] Hoogkamer, W. , S. Kipp , B. A. Spiering , and R. Kram . 2016. “Altered Running Economy Directly Translates to Altered Distance‐Running Performance.” Medicine & Science in Sports & Exercise 48, no. 11: 2175–2180. 10.1249/MSS.0000000000001012. 27327023

[ejsc70266-bib-0061] Hunter, I. , A. McLeod , D. Valentine , T. Low , J. Ward , and R. Hager . 2019. “Running Economy, Mechanics, and Marathon Racing Shoes.” Journal of Sports Sciences 37, no. 20: 2367–2373. 10.1080/02640414.2019.1633837. 31223054

[ejsc70266-bib-0062] Jones, A. M. 2024. “The Fourth Dimension: Physiological Resilience as an Independent Determinant of Endurance Exercise Performance.” Journal of Physiology 602, no. 17: 4113–4128. 10.1113/JP284205. 37606604

[ejsc70266-bib-0063] Joubert, D. P. , T. A. Dominy , and G. T. Burns . 2023. “Effects of Highly Cushioned and Resilient Racing Shoes on Running Economy at Slower Running Speeds.” International Journal of Sports Physiology and Performance 18, no. 2: 164–170. 10.1123/ijspp.2022-0227. 36626911

[ejsc70266-bib-0064] Joubert, D. P. , and G. P. Jones . 2022. “A Comparison of Running Economy Across Seven Highly Cushioned Racing Shoes With Carbon‐Fibre Plates.” Footwear Science 14, no. 2: 71–83. 10.1080/19424280.2022.2038691.

[ejsc70266-bib-0065] Joubert, D. P. , G. M. Oehlert , E. J. Jones , and G. T. Burns . 2024. “Comparative Effects of Advanced Footwear Technology in Track Spikes and Road‐Racing Shoes on Running Economy.” International Journal of Sports Physiology and Performance 19, no. 7: 705–711. 10.1123/ijspp.2023-0372. 38815961

[ejsc70266-bib-0066] Joyner, M. J. 1991. “Modeling: Optimal Marathon Performance on the Basis of Physiological Factors.” Journal of Applied Physiology 70, no. 2: 683–687. 10.1152/jappl.1991.70.2.683. 2022559

[ejsc70266-bib-0067] Kerdok, A. E. , A. A. Biewener , T. A. McMahon , P. G. Weyand , and H. M. Herr . 2002. “Energetics and Mechanics of Human Running on Surfaces of Different Stiffnesses.” Journal of Applied Physiology 92, no. 2: 469–478. 10.1152/japplphysiol.01164.2000. 11796653

[ejsc70266-bib-0068] Kiesewetter, P. , S. Bräuer , R. Haase , N. Nitzsche , C. Mitschke , and T. L. Milani . 2022. “Do Carbon‐Plated Running Shoes With Different Characteristics Influence Physiological and Biomechanical Variables During a 10 km Treadmill Run?” Applied Sciences 12, no. 15: 7949. 10.3390/app12157949.

[ejsc70266-bib-0069] Kim, H. , and J. Ahn . 2025. “Technologically Advanced Running Shoes Reduce Biomechanical Factors of Running Related Injury Risk.” Scientific Reports 15, no. 1: 17828. 10.1038/s41598-025-03029-0. PMC12098673 40404916

[ejsc70266-bib-0070] Kipp, S. , R. Kram , and W. Hoogkamer . 2019. “Extrapolating Metabolic Savings in Running: Implications for Performance Predictions.” Frontiers in Physiology 10, no. 79: 79. 10.3389/fphys.2019.00079. PMC6378703 30804807

[ejsc70266-bib-0071] Kirby, B. S. , E. Hughes , M. Haines , S. Stinman , and B. J. Winn . 2019. “Influence of Performance Running Footwear on Muscle Soreness and Damage.” Supplement, Footwear Science 11, no. S1: S188–S189. 10.1080/19424280.2019.1606325.

[ejsc70266-bib-0072] Knopp, M. , B. Muniz‐Pardos , H. Wackerhage , et al. 2023. “Variability in Running Economy of Kenyan World‐Class and European Amateur Male Runners With Advanced Footwear Running Technology: Experimental and Meta‐Analysis Results.” Sports Medicine 53, no. 6: 1255–1271. 10.1007/s40279-023-01816-1. PMC10185608 36862339

[ejsc70266-bib-0073] Kram, R. 2022. “Ergogenic Distance Running Shoes: How Do We Think They Work and How Can We Understand Them Better?” Footwear Science 14, no. 3: 139–146. 10.1080/19424280.2022.2127545.

[ejsc70266-bib-0074] Kuzmeski, J. , M. Bertschy , L. Healey , Z. Barrons , and W. Hoogkamer . 2025. “Data Driven Shoe Design Improves Running Economy Beyond State‐of‐the‐Art Advanced Footwear Technology Running Shoes.” bioRxiv. 10.1101/2025.04.13.648601. PMC13262284 41831565

[ejsc70266-bib-0075] Langley, J. O. , H. R. Branthwaite , N. Chockalingam , and J. J. Forsyth . 2023. “Determining the Effect and Magnitude of Advanced Footwear Technology on Female Distance Running Performance.” Footwear Science 15, no. 3: 161–169. 10.1080/19424280.2023.2219651.

[ejsc70266-bib-0076] Lee, R. Y. , H. Noro , K. Hata , Y. Yamazaki , and T. Yanagiya . 2026. “T2‐Weighted MR Imaging of Thigh Muscle Response in Male Distance Runners Wearing Advanced Footwear Technology.” Gait & Posture 127: 110168. 10.1016/j.gaitpost.2026.110168. 41905320

[ejsc70266-bib-0077] Li, F. , D. Sun , C. Zhu , et al. 2026. “The Impact of Toe Spring and Foot Strike Angle on Forefoot Running Biomechanics: A Finite Element Analysis.” Computer Methods in Biomechanics and Biomedical Engineering 29, no. 3: 544–554. 10.1080/10255842.2024.2402860. 39282741

[ejsc70266-bib-0078] Lieberman, D. E. , A. G. Warrener , J. Wang , and E. R. Castillo . 2015. “Effects of Stride Frequency and Foot Position at Landing on Braking Force, Hip Torque, Impact Peak Force and the Metabolic Cost of Running in Humans.” Journal of Experimental Biology 218, no. Pt 21: 3406–3414. 10.1242/jeb.125500. 26538175

[ejsc70266-bib-0079] Lin S. , H. Yuan , J. Shao , et al. 2026. “Effects of Shoe Longitudinal Bending Stiffness and Runner Level on Achilles Tendon Loading in Experienced and Novice Male Runners: A Musculoskeletal Modelling Study.” 10.21203/rs.3.rs-9811102/v1.

[ejsc70266-bib-0080] Lippa, N. M. , P. K. Collins , J. Bonacci , S. G. Piland , J. W. Rawlins , and T. E. Gould . 2017. “Mechanical Ageing Performance of Minimalist and Traditional Footwear Foams.” Footwear Science 9, no. 1: 9–20. 10.1080/19424280.2016.1228702.

[ejsc70266-bib-0081] Liu, T. , E. S. Matijevich , Z. Chu , F. Yang , and B. Nigg . 2024. “Role of Midsole Hollow Structure in Energy Storage and Return in Running Shoes.” Journal of Biomechanics 171: 112197. 10.1016/j.jbiomech.2024.112197. 38905927

[ejsc70266-bib-0082] Lyons, S. M. , M. R. Paquette , T. Tenbroek , and E. Steiner . 2025. “Effects of Advanced Midsole Foam in a Daily Trainer on Muscle Soreness, Fatigue and Training Load During Running Training.” Footwear Science 18, no. 1: 27–36. 10.1080/19424280.2025.2579974.

[ejsc70266-bib-0083] Madsen, L. L. , K. Abel , A. A. Hansen , et al. 2025. “Persistent Improvements in Running Economy With Advanced Footwear Technology During Prolonged Running in Trained Male Runners.” Scandinavian Journal of Medicine & Science in Sports 35, no. 10: e70139. 10.1111/sms.70139. PMC12464892 41001734

[ejsc70266-bib-0084] Mai, P. , L. Robertz , M. Thelen , et al. 2021. “A Method to Quantify Stiffness Across the Entire Surface of a shoe‘s Midsole.” Footwear Science 13, no. 2: 105–116. 10.1080/19424280.2021.1878286.

[ejsc70266-bib-0085] Malisoux, L. , N. Delattre , A. Urhausen , and D. Theisen . 2020. “Shoe Cushioning Influences the Running Injury Risk According to Body Mass: A Randomized Controlled Trial Involving 848 Recreational Runners.” American Journal of Sports Medicine 48, no. 2: 473–480. 10.1177/0363546519892578. 31877062

[ejsc70266-bib-0086] Malisoux, L. , P. Gette , A. Backes , N. Delattre , J. Cabri , and D. Theisen . 2021. “Relevance of Frequency‐Domain Analyses to Relate Shoe Cushioning, Ground Impact Forces and Running Injury Risk: A Secondary Analysis of a Randomized Trial With 800+ Recreational Runners.” Frontiers in Sports and Active Living 3: 744658. 10.3389/fspor.2021.744658. PMC8632264 34859204

[ejsc70266-bib-0087] Malisoux, L. , J. Ramesh , R. Mann , R. Seil , A. Urhausen , and D. Theisen . 2015. “Can Parallel Use of Different Running Shoes Decrease Running‐Related Injury Risk?” Scandinavian Journal of Medicine & Science in Sports 25, no. 1: 110–115. 10.1111/sms.12154. 24286345

[ejsc70266-bib-0088] Malisoux, L. , and D. Theisen . 2020. “Can the “Appropriate” Footwear Prevent Injury in Leisure‐Time Running? Evidence Versus Beliefs.” Journal of Athletic Training 55, no. 12: 1215–1223. 10.4085/1062-6050-523-19. PMC7740063 33064799

[ejsc70266-bib-0089] Margaria, R. 1976. Biomechanics and Energetics of Muscular Exercise. Claredon Press.

[ejsc70266-bib-0090] Maruo, Y. , and K. Takezawa . 2026. “The Impact of Advanced Footwear Technology on Running Performance and Pacing in World Marathon Majors.” Frontiers in Physiology 17: 1800107. 10.3389/fphys.2026.1800107. PMC13212062 42212262

[ejsc70266-bib-0091] Matijevich, E. S. , L. M. Branscombe , L. R. Scott , and K. E. Zelik . 2019. “Ground Reaction Force Metrics Are Not Strongly Correlated With Tibial Bone Load When Running Across Speeds and Slopes: Implications for Science, Sport and Wearable Tech.” PLoS One 14, no. 1: e0210000. 10.1371/journal.pone.0210000. PMC6336327 30653510

[ejsc70266-bib-0092] Matijevich, E. S. , J. R. Fletcher , and B. M. Nigg . 2023. “Footwear Energy Return Is Intuitively ‘Good’, But Should Not be Considered in Isolation.” Footwear Science 15, no. 2: 71–76. 10.1080/19424280.2023.2180674.

[ejsc70266-bib-0093] Matijevich, E. S. , E. C. Honert , Y. Fan , G. Lam , and B. M. Nigg . 2022. “A Foot and Footwear Mechanical Power Theoretical Framework: Towards Understanding Energy Storage and Return in Running Footwear.” Journal of Biomechanics 141: 111217. 10.1016/j.jbiomech.2022.111217. 35841784

[ejsc70266-bib-0094] Matijevich, E. S. , E. C. Honert , F. Yang , W.‐K. Lam , and B. M. Nigg . 2025. “Greater Foot and Footwear Mechanical Work Associated With Less Ankle Joint Work During Running.” Sports Biomechanics 24, no. 6: 1495–1513. 10.1080/14763141.2023.2296916. 38164950

[ejsc70266-bib-0095] Matties, J. R. , J. D. Kerr , and K. M. Rowley . 2024. “Footwear‐Specific Biomechanical and Energetic Responses to 8 Weeks of Training in Advanced Footwear Technology.” bioRxiv. 10.1101/2024.11.20.624520.

[ejsc70266-bib-0096] Matties, J. R. , and K. M. Rowley . 2025. “Sex‐Specific Metabolic and Biomechanical Responses to Advanced Footwear Technology.” Footwear Science 17, no. 3: 153–165. 10.1080/19424280.2025.2535284.

[ejsc70266-bib-0097] Matties, J. R. , and K. M. Rowley . 2026. “Cross‐Country Runners With Previous Experience With Advanced Footwear Technology Demonstrate Greater Performance Benefits.” Footwear Science 18, no. 2: 133–141. 10.1080/19424280.2026.2637938.

[ejsc70266-bib-0098] Meardon, S. A. , J. D. Willson , T. W. Kernozek , A. H. Duerst , and T. R. Derrick . 2018. “Shoe Cushioning Affects Lower Extremity Joint Contact Forces During Running.” Footwear Science 10, no. 2: 109–117. 10.1080/19424280.2018.1501771.

[ejsc70266-bib-0099] Milner, P. , C. R. Firminger , S. Nigg , B. M. Nigg , and W. B. Edwards . 2025. “Metatarsal Loads During a Prolonged Run in Advanced Footwear Technology.” Supplement, Footwear Science 17, no. S1: S106–S107. 10.1080/19424280.2025.2493283.

[ejsc70266-bib-0100] Miyazaki, T. , T. Aimi , Y. Yamada , and Y. Nakamura . 2024. “Curved Carbon Plates Inside Running Shoes Modified Foot and Shank Angular Velocity Improving Mechanical Efficiency at the Ankle Joint.” Journal of Biomechanics 172: 112224. 10.1016/j.jbiomech.2024.112224. 38971114

[ejsc70266-bib-0101] Molmen, K. S. , N. W. Almquist , and O. Skattebo . 2025. “Effects of Exercise Training on Mitochondrial and Capillary Growth in Human Skeletal Muscle: A Systematic Review and Meta‐Regression.” Sports Medicine 55, no. 1: 115–144. 10.1007/s40279-024-02120-2. PMC11787188 39390310

[ejsc70266-bib-0102] Muniz‐Pardos, B. , K. Angeloudis , I. Zelenkova , et al. 2025. “Advanced Footwear Technology in Well‐Trained Athletes: Methodological Insights From Outdoor Running.” Frontiers in Physiology 16: 1713902. 10.3389/fphys.2025.1713902. PMC12707015 41409562

[ejsc70266-bib-0103] Nahan, K. , S. Willwacher , J. Kerr , M. Héroux , and K. A. McDonald . 2025. “Positive Joint Work Redistribution in Running: The Role of Plantar Flexor Fatigue and the Effect of Advanced Footwear Technology.” bioRxiv. 10.1101/2025.10.26.684644. 42704772

[ejsc70266-bib-0104] Needles, B. J. , and A. M. Grabowski . 2024. “Does Running Speed Affect the Performance Improvements Experienced by Elite Distance Runners Wearing Advanced Footwear Technology Spikes?” Journal of Applied Physiology 137, no. 6: 1690–1698. 10.1152/japplphysiol.00610.2024. 39561014

[ejsc70266-bib-0105] Needles, B. J. , S. Kim , and A. M. Grabowski . 2026. “The Running Economy and Biomechanics of Competitive Distance Runners Wearing Advanced Footwear Technology Spikes.” European Journal of Applied Physiology, Ahead of Print. 126, no. 8: 4507–4519. 10.1007/s00421-026-06257-y. PMC13451222 42191900

[ejsc70266-bib-0106] Nielsen, A. , J. Franch , C. Heyde , M. de Zee , U. Kersting , and R. G. Larsen . 2022. “Carbon Plate Shoes Improve Metabolic Power and Performance in Recreational Runners.” International Journal of Sports Medicine 43, no. 9: 804–810. 10.1055/a-1776-7986. 35523201

[ejsc70266-bib-0107] Nielsen, A. , C. Heyde , M. B. Simonsen , et al. 2022. “Reliability of Running Economy Measurements: Influence of Shoe Familiarisation.” International Journal of Sports Medicine 43, no. 13: 1113–1117. 10.1055/a-1820-7023. 35926512

[ejsc70266-bib-0108] Ortega, J. A. , L. A. Healey , W. Swinnen , and W. Hoogkamer . 2021. “Energetics and Biomechanics of Running Footwear With Increased Longitudinal Bending Stiffness: A Narrative Review.” Sports Medicine 51, no. 5: 873–894. 10.1007/s40279-020-01406-5. 33830444

[ejsc70266-bib-0109] Paradisis, G. P. , E. Zacharogiannis , A. Bissas , and B. Hanley . 2023. “Recreational Runners Gain Physiological and Biomechanical Benefits From Super Shoes at Marathon Paces.” International Journal of Sports Physiology and Performance 18, no. 12: 1420–1426. 10.1123/ijspp.2023-0115. 37734742

[ejsc70266-bib-0110] Perrin, T. P. , R. Gerey , C. Y. M. Morio , et al. 2025. “Effect of Footwear Longitudinal Bending Stiffness on Energy Cost, Biomechanics, and Fatigue During a Treadmill Half‐Marathon.” Medicine & Science in Sports & Exercise 57, no. 4: 657–667. 10.1249/MSS.0000000000003599. 39501476

[ejsc70266-bib-0111] Perry, D. , H. Lino , M. Bertschy , and W. Hoogkamer . 2025. “Effects of Longitudinal Bending Stiffness and Midsole Foam on Running Energetics.” Footwear Science 17, no. 1: 3–9. 10.1080/19424280.2024.2431004.

[ejsc70266-bib-0112] Petrella, D. , W. Swinnen , N. Tam , B. E. N. Lane , and B. E. N. E. D. I. C. T. E. Vanwanseele . 2026. “The Isolated Effect of Midsole Compliance on Running Economy and Biomechanics in Highly Trained Runners.” Medicine & Science in Sports & Exercise 58, no. 2: 310–320. 10.1249/MSS.0000000000003864. 41090637

[ejsc70266-bib-0113] Qian, Y. , Z. Xia , Y. Song , et al. 2026. “Region‐Specific Plantar Fascia Stress Responses to Carbon‐Fiber Plate Stiffness During Running: A Dynamic Explicit Finite Element Analysis.” Journal of Bionic Engineering: 1–18. 10.1007/s42235-026-00983-x.

[ejsc70266-bib-0114] Riddick, R. C. , and A. D. Kuo . 2022. “Mechanical Work Accounts for Most of the Energetic Cost in Human Running.” Scientific Reports 12, no. 1: 645. 10.1038/s41598-021-04215-6. PMC8755824 35022431

[ejsc70266-bib-0115] Riedl, M. , C. von Diecken , and O. Ueberschär . 2024. “One Shoe to Fit Them All? Effect of Various Carbon Plate Running Shoes on Running Economy in Male and Female Amateur Triathletes and Runners at Individual Training and Race Paces.” Applied Sciences 14, no. 24: 11535. 10.3390/app142411535.

[ejsc70266-bib-0116] Rodrigo‐Carranza, V. , A. Alda‐Blanco , J. Kuzmeski , F. González‐Mohíno , R. Agüir Anguita , and W. Hoogkamer . 2026. “Impact of Running Shoe Midsole Properties on Energy Cost of Running: Insights Into Midsole Stiffness and Resilience Properties.” International Journal of Sports Physiology and Performance 21, no. 6: 708–714. 10.1123/ijspp.2025-0475. 41765002

[ejsc70266-bib-0117] Rodrigo‐Carranza, V. , F. Gonzalez‐Mohino , J. Santos Del Cerro , J. Santos‐Concejero , and J. M. González‐Ravé . 2021. “Influence of Advanced Shoe Technology on the Top 100 Annual Performances in Men's Marathon From 2015 to 2019.” Scientific Reports 11, no. 1: 22458. 10.1038/s41598-021-01807-0. PMC8599511 34789828

[ejsc70266-bib-0118] Rodrigo‐Carranza, V. , F. Gonzalez‐Mohino , J. Santos‐Concejero , and J. M. Gonzalez‐Rave . 2022. “Impact of Advanced Footwear Technology on Elite Men's in the Evolution of Road Race Performance.” Journal of Sports Sciences 40, no. 23: 2661–2668. 10.1080/02640414.2023.2183103. 36814065

[ejsc70266-bib-0119] Rodrigo‐Carranza, V. , W. Hoogkamer , J. M. Gonzalez‐Rave , and F. Gonzalez‐Mohino . 2024. “Relationship Between Advanced Footwear Technology Longitudinal Bending Stiffness and Energy Cost of Running.” Scandinavian Journal of Medicine & Science in Sports 34, no. 6: e14687. 10.1111/sms.14687. 38923087

[ejsc70266-bib-0120] Rodrigo‐Carranza, V. , W. Hoogkamer , J. M. González‐Ravé , et al. 2024. “Influence of Different Midsole Foam in Advanced Footwear Technology Use on Running Economy and Biomechanics in Trained Runners.” Scandinavian Journal of Medicine & Science in Sports 34, no. 1: e14526. 10.1111/sms.14526. 37858294

[ejsc70266-bib-0121] Rodrigo‐Carranza, V. , V. Munoz de la Cruz , and W. Hoogkamer . 2025. “Influence of advanced‐footwear‐technology Spikes on Middle‐ and Long‐Distance Running Performance Measures in Trained Runners.” International Journal of Sports Physiology and Performance 20, no. 5: 689–699. 10.1123/ijspp.2024-0377. 40088898

[ejsc70266-bib-0122] Ryan, M. , and E. M. Farina . 2025. “Does Advanced Footwear Technology Cause or Protect Against Injuries? a 12‐Week Prospective Investigation in 195 Half‐Marathon Runners.” Supplement, Footwear Science 17, no. S1: S246–S247. 10.1080/19424280.2025.2492279.

[ejsc70266-bib-0123] Scherrer, D. , K. A. Legg , C. W. Rogers , J. S. Gottschall , and D. J. Cochrane . 2026. “Mechanical Testing of Midsole Properties at Different Impact Frequencies.” Applied Sciences 16, no. 10: 5130. 10.3390/app16105130.

[ejsc70266-bib-0124] Schwalm, L. C. , D. Fohrmann , M. Schaffarczyk , T. Gronwald , S. Willwacher , and K. Hollander . 2024. “Habituation Does Not Change Running Economy in Advanced Footwear Technology.” International Journal of Sports Physiology and Performance 19, no. 11: 1285–1290. 10.1123/ijspp.2024-0136. 39187239

[ejsc70266-bib-0125] Schwalm, L. C. , D. Fohrmann , M. Schaffarczyk , A. Herrmann , T. Gronwald , and K. Hollander . 2025. “Running Economy Benefits of Advanced Footwear Technologies Remain Over a Prolonged Run in Highly Trained Distance Runners‐A Randomized Crossover Trial.” Medicine & Science in Sports & Exercise 57, no. 12: 2919–2928. 10.1249/MSS.0000000000003823. 40762986

[ejsc70266-bib-0126] Scott, S. H. , and D. A. Winter . 1990. “Internal Forces of Chronic Running Injury Sites.” Medicine & Science in Sports & Exercise 22, no. 3: 357–369. 10.1249/00005768-199006000-00013. 2381304

[ejsc70266-bib-0127] Seglina, I. , K. Torniainen , L. Kvarforth , S. Hallström , and A. Arndt . 2026. “Sex and Isolated Anthropometric Measures Do Not Explain Individual Differences in Responsiveness to Advanced Footwear Technology in Highly Trained Runners.” Scandinavian Journal of Medicine & Science in Sports 36, no. 2: e70234. 10.1111/sms.70234. PMC12926519 41725050

[ejsc70266-bib-0128] Sobhani, S. , S. Bredeweg , R. Dekker , et al. 2014. “Rocker Shoe, Minimalist Shoe, and Standard Running Shoe: A Comparison of Running Economy.” Journal of Science and Medicine in Sport 17, no. 3: 312–316. 10.1016/j.jsams.2013.04.015. 23711621

[ejsc70266-bib-0129] Song, Y. , X. Cen , H. Chen , et al. 2023. “The Influence of Running Shoe With Different Carbon‐Fiber Plate Designs on Internal Foot Mechanics: A Pilot Computational Analysis.” Journal of Biomechanics 153: 111597. 10.1016/j.jbiomech.2023.111597. 37126883

[ejsc70266-bib-0130] Song, Y. , X. Cen , D. Sun , et al. 2024. “Curved Carbon‐Plated Shoe May Further Reduce Forefoot Loads Compared to Flat Plate During Running.” Scientific Reports 14, no. 1: 13215. 10.1038/s41598-024-64177-3. PMC11162459 38851842

[ejsc70266-bib-0131] Steele, J. , L. VanKeersbilck , J. B. Ward , and I. Hunter . 2026. “Advanced Footwear Technology Slows Oxygen Uptake Drift and Reduces Neuromuscular Fatigue During Extended Running.” International Journal of Sports Physiology and Performance 21, no. 1: 65–71. 10.1123/ijspp.2025-0218. 41207296

[ejsc70266-bib-0132] Stephen, C. H. N. , L. A. Kelly , R. W. Schuster , and L. E. Diamond . 2025. “The Effects of Running Shoe Longitudinal Bending Stiffness and Midsole Energy Return on Oxygen Consumption and Ankle Mechanics and Energetics: A Systematic Review and Meta‐Analysis.” Journal of Sport and Health Science 14: 101069. 10.1016/j.jshs.2025.101069. PMC12305619 40550392

[ejsc70266-bib-0133] Subramanium, A. , J. Vienneau , S. R. Nigg , and B. M. Nigg . 2024. “A Methodological Proof‐of‐Concept of the Teeter‐Totter Effect.” Journal of Sports Sciences 42, no. 15: 1432–1438. 10.1080/02640414.2024.2394746. 39207092

[ejsc70266-bib-0134] Swinnen, W. , W. Hoogkamer , F. De Groote , and B. Vanwanseele . 2023. “Faster Triceps Surae Muscle Cyclic Contractions Alter Muscle Activity and Whole Body Metabolic Rate.” Journal of Applied Physiology 134, no. 2: 395–404. 10.1152/japplphysiol.00575.2022. 36603047

[ejsc70266-bib-0135] Tankink, T. , H. Houdijk , and J. M. Hijmans . 2024. “Human‐in‐the‐Loop Optimized Rocker Profile of Running Shoes to Enhance Ankle Work and Running Economy.” European Journal of Sport Science 24, no. 1: 164–173. 10.1002/ejsc.12054.

[ejsc70266-bib-0136] Trama, R. , J. W. Wannop , E. Smith , and D. J. Stefanyshyn . 2023. “The Influence of Midsole Horizontal and Vertical Deformation on Soft Tissue Vibrations and Bone Acceleration During Running.” Journal of Sports Sciences 41, no. 11: 1047–1055. 10.1080/02640414.2023.2259208. 37724576

[ejsc70266-bib-0137] Tung, K. D. , J. R. Franz , and R. Kram . 2014. “A Test of the Metabolic Cost of Cushioning Hypothesis During Unshod and Shod Running.” Medicine & Science in Sports & Exercise 46, no. 2: 324–329. 10.1249/MSS.0b013e3182a63b81. 24441213

[ejsc70266-bib-0138] Van Hooren, B. , R. Copier , S. Pedersen , Z. Balamouti , and K. Meijer . 2025. “The Mediating Effect of Running Biomechanics, Anthropometrics, Muscle Architecture, and Comfort on Running Economy Across Different Shoes.” Scandinavian Journal of Medicine & Science in Sports 35, no. 6: e70087. 10.1111/sms.70087. PMC12141809 40474459

[ejsc70266-bib-0139] Van Hooren, B. , J. T. Fuller , J. D. Buckley , et al. 2020. “Is Motorized Treadmill Running Biomechanically Comparable to Overground Running? A Systematic Review and Meta‐Analysis of Cross‐Over Studies.” Sports Medicine 50, no. 4: 785–813. 10.1007/s40279-019-01237-z. PMC7069922 31802395

[ejsc70266-bib-0140] Van Hooren, B. , T. Souren , F. Miqueu , and B. C. Bongers . 2026. “Technological and Biological Reliability, and Validity of Five Different CPET Systems During Simulated and Human Exercise.” Scandinavian Journal of Medicine & Science in Sports 36, no. 1: e70184. 10.1111/sms.70184. PMC12831196 41578876

[ejsc70266-bib-0141] Van Hooren, B. , L. van Rengs , and K. Meijer . 2024. “Per‐Step and Cumulative Load at Three Common Running Injury Locations: The Effect of Speed, Surface Gradient, and Cadence.” Scandinavian Journal of Medicine & Science in Sports 34, no. 2: e14570. 10.1111/sms.14570. 38389144

[ejsc70266-bib-0142] Werkhausen, A. , M. Lund‐Hansen , L. Wiedenbruch , K. Peikenkamp , and H. Rice . 2024. “Technologically Advanced Running Shoes Reduce Oxygen Cost and Cumulative Tibial Loading Per Kilometer in Recreational Female and Male Runners.” Scientific Reports 14, no. 1: 11903. 10.1038/s41598-024-62263-0. PMC11126714 38789519

[ejsc70266-bib-0143] Willwacher, S. , P. Mai , J. Helwig , M. Hipper , B. Utku , and J. Robbin . 2024. “Does Advanced Footwear Technology Improve Track and Road Racing Performance? An Explorative Analysis Based on the 100 Best Yearly Performances in the World Between 2010 and 2022.” Sports Medicine‐Open 10, no. 1: 14. 10.1186/s40798-024-00683-y. PMC10853158 38332220

[ejsc70266-bib-0144] Worobets, J. , J. W. Wannop , E. Tomaras , and D. Stefanyshyn . 2014. “Softer and More Resilient Running Shoe Cushioning Properties Enhance Running Economy.” Footwear Science 6, no. 3: 147–153. 10.1080/19424280.2014.918184.

[ejsc70266-bib-0145] Xiao, Y. , X. Hu , D. Tian , and A. Qiu . 2026. “Effects of Advanced Footwear Technology on Running Economy and Endurance Performance: A Meta‐Analysis.” International Journal of Sports Medicine 47, no. 2: 81–94. 10.1055/a-2637-7283. 40527489

[ejsc70266-bib-0146] Xu, Y. , C. Zhu , Y. Fang , et al. 2025. “The Effects of Different Carbon‐Fiber Plate Shapes in Shoes on Lower Limb Biomechanics Following Running‐Induced Fatigue.” Frontiers in Bioengineering and Biotechnology 13: 1539976. 10.3389/fbioe.2025.1539976. PMC11850346 40008032

[ejsc70266-bib-0147] Zandbergen, M. A. , X. J. Ter Wengel , R. P. van Middelaar , J. H. Buurke , P. H. Veltink , and J. Reenalda . 2025. “Peak Tibial Acceleration Should Not be Used as Indicator of Tibial Bone Loading During Running.” Sports Biomechanics 24, no. 10: 2953–2970. 10.1080/14763141.2022.2164345. 36645012

[ejsc70266-bib-0148] Zanini, M. , J. P. Folland , and R. C. Blagrove . 2026. “Regular Long Runs and Higher Training Volumes Are Associated With Better Running Economy Durability in Performance Matched Well‐Trained Male Runners.” Medicine & Science in Sports & Exercise 58, no. 1: 162–173. 10.1249/MSS.0000000000003840. 40878015

